# Longitudinal gut microbiome dynamics in neurosurgical ICU patients: *Enterococcus* expansion as a feature of microbial restructuring

**DOI:** 10.1186/s12866-026-05450-5

**Published:** 2026-08-17

**Authors:** Yangyang Guo, Jindong Zhang, Bin Zhang, Mengshi Yang, Jingwei Zhao, Guangqiang Chen, Guangzhi Shi

**Affiliations:** 1 Department of Critical Care, Beijing Tiantan Hospital, Capital Medical University, Beijing, China; 2 Department of Gastroenterology, Peking University Third Hospital, Beijing, China; 3 Beijing Key Laboratory of Human Microbiome Precision Medicine and Microbial Agent Development, Beijing, China

**Keywords:** Neurosurgical ICU, Gut microbiome, Longitudinal analysis, *Enterococcus*, Microbial restructuring

## Abstract

**Background:**

Gut microbiota alterations are commonly observed in critical illness, but their temporal dynamics in neurosurgical ICU patients remain insufficiently characterized. This study aimed to describe postoperative microbial trajectories and their clinical context during ICU stay.

**Methods:**

We conducted a longitudinal study of 39 postoperative neurosurgical ICU patients, with 101 rectal swab samples collected on ICU Days 1, 7, and 14. Microbiota profiles were generated using 16S rRNA sequencing. Temporal shifts and configurations were analyzed using diversity analyses, PERMANOVA, and OTU-level clustering. Clinical features associated with *Enterococcus* (OTU502) abundance were examined using regression models, and microbiota–clinical relationships were further explored using redundancy and variance partitioning analyses.

**Results:**

Patients were stratified into short-stay (< 14 days) and long-stay (≥ 14 days) groups. Alpha diversity showed a mild, non-significant decline by Day 7 with a slight increase at Day 14 in long-stay patients, while beta diversity demonstrated significant temporal restructuring. Genus-level analyses revealed stay-dependent changes: short-stay patients showed early loss of anaerobic genera and enrichment of *Enterococcus* by Day 7, whereas long-stay patients exhibited persistent anaerobe depletion and later emergence of *Enterococcus*. OTU clustering identified two profiles dominated by *Enterococcus* (OTU502) or *Escherichia–Shigella* (OTU967). Higher OTU502 abundance at Day 7 was more frequently observed in patients with greater illness severity and lower GCS scores. Prolonged probiotic use showed a non-significant trend toward a lower likelihood of high OTU502 abundance. Measured clinical variables accounted for a limited proportion of observed microbial variance (3–7%).

**Conclusions:**

Neurosurgical ICU patients displayed time-stratified microbial trajectories. *Enterococcus* (OTU502) enrichment represents a consistent feature of microbiota restructuring during ICU stay. These findings provide a descriptive framework for understanding microbiome dynamics in neurosurgical ICU patients.

**Trial registration:**

Chinese Clinical Trial Registry (ChiCTR2000031760). Registered on 9 April 2020.

**Supplementary Information:**

The online version contains supplementary material available at https://doi.org/10.1186/s12866-026-05450-5.

## Background

Disruption of the gut microbiota is increasingly recognized as an important feature of critical illness and has been linked to infection risk, metabolic dysfunction, and adverse outcomes in general ICU populations [1]. Studies in sepsis, respiratory failure, and multisystem organ failure consistently report a rapid decline in obligate anaerobes, an increase in oxygen-tolerant organisms, and sustained disturbance of the intestinal microbiota [2, 3]. These findings, however, are derived mainly from medical ICU cohorts and may not reflect patterns in postoperative neurosurgical patients, in whom neurological injury contributes substantially to the physiological stresses that influence gut microbiota.

The microbiota–gut–brain axis provides a bidirectional link between neural activity, autonomic regulation, and the gut microbiota [4–6]. Neurosurgical and neurocritical-care patients commonly experience sympathetic hyperactivation, neurogenic gastrointestinal dysmotility, impaired mucosal perfusion, bile-acid disturbances, and systemic neuroinflammation, all of which can affect this axis [7].

Despite these characteristic disturbances, longitudinal gut microbiota dynamics in neurosurgical ICU patients remain poorly characterized, and their clinical relevance is uncertain [8–10]. Most ICU microbiome studies have relied on single or infrequent sampling, limiting understanding of whether microbial communities progressively deteriorate, partially recover, or follow heterogeneous trajectories during ICU stay [3, 11]. Although increased abundances of facultative organisms such as *Enterococcus* have been reported in ICU patients [12–14], it remains unclear whether this reflects general critical illness-associated microbiota disruption or has relevance in the neuro-ICU setting [8–10].

To address these gaps, we conducted a longitudinal microbiome study in neurosurgical ICU patients, integrating serial microbiota sampling with detailed clinical phenotyping across early and prolonged ICU phases. Our objectives were to describe time-dependent changes in the gut microbiota, identify clinical and treatment-related factors associated with these patterns, and examine microbial features in relation to clinical characteristics during ICU stay.

## Methods

### Study design and patient enrollment

**Fig. 1:**
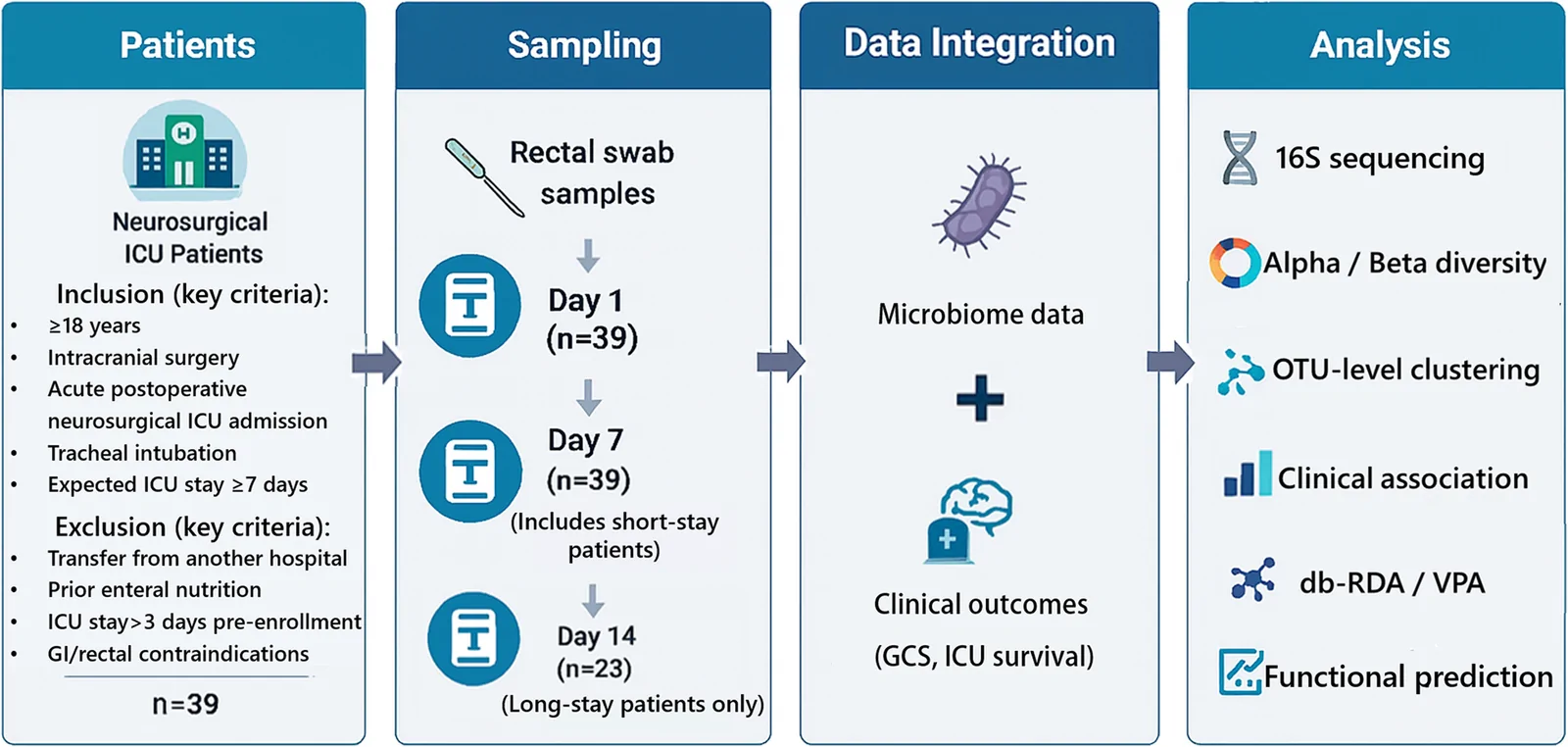
Overview of the study design and methodology. This figure outlines the overall workflow of the study, including patient enrollment, the timing of rectal swab collection on ICU Days 1, 7, and 14, and how microbiome data were combined with clinical information. The downstream analyses are also summarized, covering diversity measurements, OTU502-related stratification, db-RDA and VPA assessments, and functional prediction using PICRUSt2 and BugBase

This study was based on a prospective cohort conducted in the Department of Critical Care at Beijing Tiantan Hospital, Capital Medical University. The study was approved by the Institutional Review Board of Beijing Tiantan Hospital (KY2019-077-03) and registered in the Chinese Clinical Trial Registry (ChiCTR2000031760). Patient recruitment was conducted between May 2020 and December 2021. Using this cohort, we examined gut microbial alterations and their clinical associations in neurosurgical ICU patients after intracranial surgery (Fig. 1).

Patients were eligible if they met all the following criteria: age ≥ 18 years, in an acute stress stage after neurosurgery or intracranial trauma, receiving tracheal intubation, and expected to receive ICU treatment for ≥ 7 days. Exclusion criteria were: transfer from another hospital; prior enteral nutrition before enrollment; ICU stay > 3 days before inclusion; contraindications to enteral feeding (e.g., intestinal obstruction, gastrointestinal bleeding, or prone position); prior gastrostomy or jejunostomy; or history of rectal surgery or perianal infection.

Because spontaneous defecation is often absent in postoperative neurosurgical ICU patients, rectal swabs were used as an alternative to fecal samples for microbiome sampling [15–17]. Previous studies have shown that rectal swabs can yield microbial community profiles broadly comparable to fecal samples in 16 S rRNA sequencing analyses [18, 19].

For the longitudinal microbiome analyses, patients were required to have rectal swab samples collected at predefined timepoints (Day 1 and Day 7 for all patients, and Day 14 for those remaining in the ICU), along with corresponding clinical data. A total of 39 patients met these criteria and were included in the final analysis.

Patients were stratified by ICU length of stay (LOS) into short-stay (< 14 days, *n* = 16) and long-stay (≥ 14 days, *n* = 23) groups. ICU LOS was defined as the duration from ICU admission to transfer or death. Demographic, perioperative, medication, and complication data were prospectively recorded. Systemic antibiotics administered in this cohort included cefuroxime, ceftazidime, ceftriaxone, piperacillin/tazobactam, cefoperazone/sulbactam, linezolid, vancomycin, and meropenem. Meropenem, piperacillin/tazobactam, and cefoperazone/sulbactam were classified as providing anti-anaerobic coverage, whereas cefuroxime, ceftazidime, ceftriaxone, linezolid, and vancomycin were not classified as providing anti-anaerobic coverage. Probiotic use, when administered, consisted of live *Bacillus licheniformis* capsules and *Bifidobacterium* powder, administered three times daily at standard ICU doses according to routine clinical practice.

### Sample collection and DNA extraction

Rectal swabs were collected by inserting the swab 2–3 cm into the anal canal and rotating gently to avoid perianal contamination, following standard clinical microbiology sampling procedures. After collection, rectal swabs were immediately placed in RNAlater^®^ (Thermo Fisher Scientific, USA) for stabilization, stored at − 80 °C within 2 h, and transported on dry ice for DNA extraction. Microbial DNA was extracted using the DNeasy PowerSoil Pro Kit (Qiagen, Germany) following the manufacturer’s protocol. DNA integrity was confirmed by 1% agarose gel electrophoresis, and concentration and purity were determined using a NanoDrop ND-2000 spectrophotometer (Thermo Fisher Scientific, USA). DNA purity was further verified by an A260/A230 ratio > 1.5 and a minimum concentration ≥ 10 ng/µL, confirming suitability for amplification. Samples with A260/A280 ratios between 1.8 and 2.0 were used for sequencing.

### 16S rRNA gene amplification and sequencing

The V3–V4 regions of the bacterial 16S rRNA gene were amplified using primers 338F (5′-ACTCCTACGGGAGGCAGCAG-3′) and 806R (5′-GGACTACHVGGGTWTCTAAT-3′). PCR reactions were performed in triplicate 20 µL systems containing 4 µL 5× FastPfu Buffer, 2 µL 2.5 mM dNTPs, 0.8 µL of each primer (5 µM), 0.4 µL FastPfu Polymerase (TransGen Biotech, China; Cat# AP221-02), and 10 ng template DNA, with nuclease-free water added to a final 20 µL. Thermal cycling comprised 95 °C for 3 min, 27 cycles of 95 °C for 30 s, 55 °C for 30 s, 72 °C for 45 s, and a final extension at 72 °C for 10 min. PCR products were verified by 2% agarose gel electrophoresis for target bands (~ 460 bp); only bands of ~ 460 bp were excised and purified using an AxyPrep DNA Gel Extraction Kit (Axygen Biosciences, USA), followed by quantification using Qubit fluorometry (Thermo Fisher Scientific, USA). Purified amplicons were pooled in equimolar amounts based on Qubit quantification and sequenced on an Illumina MiSeq platform (2 × 300 bp paired-end) at Majorbio Bio-Pharm Technology Co., Ltd. (Shanghai, China).

### Bioinformatic processing and OTU construction

Raw reads were demultiplexed, quality-filtered with *fastp* v0.19.6, and merged using *FLASH* v1.2.11 (overlap > 10 bp, mismatch ≤ 0.2). High-quality reads were clustered into operational taxonomic units (OTUs) at 97% similarity using *USEARCH* v11.0, and representative sequences were taxonomically assigned with the *RDP Classifier* v11.5 against the *SILVA* v138 database (confidence ≥ 70%). Potential chloroplast and mitochondrial sequences were removed before downstream analysis. The OTU table was rarefied to 20,000 reads per sample, achieving > 99% Good’s coverage. Species-level assignments derived from SILVA v138 should be interpreted with caution, because 16S rRNA V3–V4 regions have limited resolution for distinguishing closely related taxa. Therefore, species-level annotations in this study are considered provisional, and OTUs are referred to at the genus level throughout the manuscript to avoid overinterpretation of database-based species calls.

### Microbiome statistical analysis

Alpha diversity was assessed using multiple indices, including Sobs, Chao1, ACE, Shannon, Simpson, and Pielou’s evenness. Alpha diversity indices were compared using the Wilcoxon rank-sum test for two-group comparisons and the Kruskal–Wallis test for comparisons involving more than two groups.

Beta diversity was calculated based on Bray–Curtis dissimilarity and visualized using principal coordinates analysis (PCoA). Differences in microbial community composition between groups or across time points were assessed using permutational multivariate analysis of variance (PERMANOVA) with 999 permutations.

Differentially abundant taxa were initially screened using linear discriminant analysis effect size (LEfSe), with LDA thresholds of 3.0 for within-group temporal comparisons and 2.0 for between-group comparisons at matched time points. Genus-level differential abundance was subsequently evaluated using statistical testing with false discovery rate (FDR) correction. Temporal trends of representative genera were examined descriptively across ICU Day 1, Day 7, and Day 14.

Unsupervised clustering was performed on OTU-level Bray–Curtis dissimilarities to identify reproducible microbial configurations. Hierarchical clustering with Ward linkage was applied, and cluster stability was evaluated using approximately unbiased bootstrap resampling with 1,000 iterations. Differences in clinical characteristics between microbial configurations were assessed using the Wilcoxon rank-sum test for continuous variables and Fisher’s exact test for categorical variables.

Multicollinearity among clinical covariates was assessed using the variance inflation factor (VIF). Variables with very high VIF values were examined for overlapping clinical meaning, and redundant variables were excluded when appropriate while clinically essential variables were retained. Because some residual collinearity remained among retained variables, db-RDA results were interpreted as exploratory community-level associations rather than independent effects of individual predictors. Distance-based redundancy analysis (db-RDA) and variation partitioning analysis (VPA) were used to describe the overall variance in microbial community structure explained by the selected clinical factors. Correlations between dominant genera and clinical parameters were evaluated using Spearman’s rank correlation and visualized as heatmaps. Functional prediction was performed using PICRUSt2, and microbial phenotypes were inferred using BugBase.

### Analyses of OTU502 abundance and clinical features

Analyses were performed in the full cohort unless otherwise specified. For analyses involving recovery-related clinical measures, only patients who survived to ICU discharge were included. Clinical features associated with high OTU502 abundance were examined using multivariable logistic regression models. Patients were stratified into high- and low-OTU502 groups according to the median OTU502 relative abundance at ICU Day 7. The median was used as an exploratory, distribution-based cut-off because no pre-specified threshold for OTU502 abundance was available in this study.

To account for repeated measurements of OTU502 abundance over time, linear mixed-effects models (LMMs) were constructed using serial OTU502 measurements obtained at ICU Day 1, Day 7, and Day 14, with patient ID included as a random intercept. OTU502 relative abundance was log-transformed after adding a pseudocount of 1, and fixed effects included timepoint, duration of probiotic use, and duration of anaerobic antibiotic coverage.

To characterize temporal changes in OTU502 abundance, patient-specific OTU502 trajectories were summarized as the slope derived from linear regression of log-transformed OTU502 abundance across ICU Day 1, Day 7, and Day 14. These trajectory metrics were then examined in relation to clinical measures using linear regression models for continuous variables and logistic regression models for binary variables.

All statistical tests were two-tailed. For analyses involving multiple comparisons, p values were adjusted using the Benjamini–Hochberg FDR method, and an FDR-corrected q value < 0.05 was considered statistically significant. All statistical analyses were performed on the Majorbio Cloud Platform and in R (v3.6.1) using the vegan, psych, ggplot2, and lme4 packages.

### Ethical approval

The study was approved by the Institutional Review Board of Beijing Tiantan Hospital (KY2019-077-03), and written informed consent was obtained from the legal representatives of all participants.

## Results

### Baseline characteristics of the study population

A total of 39 patients who underwent intracranial surgery were included, contributing 101 rectal swab samples collected at serial time points during their ICU stay. According to the ICU length of stay (LOS), patients were stratified into a short-stay group (< 14 days, *n* = 16) and a long-stay group (≥ 14 days, *n* = 23). The baseline demographic and clinical characteristics of both cohorts are summarized in Table 1. Overall, the two groups were comparable in demographic variables, comorbidities, and intraoperative parameters. A higher proportion of male patients was observed in the long-stay group, although this difference did not reach statistical significance. However, patients with prolonged ICU stays tended to have higher illness severity and showed a greater incidence of postoperative complications, along with longer exposure to antibiotic, probiotic, and proton pump inhibitor (PPI) therapies.

**Table 1:** Baseline demographic and clinical characteristics of patients stratified by ICU length of stay (LOS)

Variable	Total (*n* = 39)	ICU LOS < 14 days (*n* = 16)	ICU LOS ≥ 14 days (*n* = 23)	*p*-value
Demographics				
Sex (male), n (%)	20 (51.3)	5 (31.3)	15 (65.2)	0.054
Age, years	45.8 ± 15.2	50.1 ± 11.3	42.9 ± 17.0	0.150
BMI, kg/m²	24.0 ± 3.3	24.8 ± 3.8	23.5 ± 2.8	0.240
Hypertension, n (%)	12 (30.8)	4 (25.0)	8 (34.8)	0.720
Diabetes mellitus, n (%)	5 (12.8)	2 (12.5)	3 (13.0)	0.990
Clinical Diagnosis and Surgery				
Admitting diagnosis (Supratentorial / Infratentorial / Other), n (%)	10 (25.6) / 25 (64.1) / 4 (10.3)	4 (25.0) / 10 (62.5) / 2 (12.5)	6 (26.1) / 15 (65.2) / 2 (8.7)	0.890
Operative duration, h	7.1 ± 3.0	6.2 ± 2.0	7.7 ± 3.4	0.120
Estimated blood loss, mL	1286 ± 2244	1144 ± 2464	1385 ± 2130	0.750
Intraoperative hypotension duration, min	11.4 ± 23.3	7.8 ± 11.1	13.9 ± 29.0	0.430
Postoperative complications, n (%)	12 (30.8)	1 (6.3)	11 (47.8)	0.012^*^
Postoperative hypoalbuminemia (Normal / Mild / Moderate / Severe), n (%)	22 (56.4) / 12 (30.8) / 3 (7.7) / 2 (5.1)	9 (56.3) / 4 (25.0) / 2 (12.5) / 1 (6.3)	13 (56.5) / 8 (34.8) / 1 (4.3) / 1 (4.3)	0.820
Severity and Neurological Scores				
APACHE II score at ICU admission	14.8 ± 4.4	13.9 ± 4.4	15.5 ± 4.4	0.270
Lowest GCS during ICU stay	8.6 ± 2.4	9.1 ± 2.3	8.2 ± 2.5	0.290
GCS score at discharge	11.6 ± 3.8	12.4 ± 3.7	11.0 ± 3.8	0.240
Therapies and ICU Exposure				
Vasopressor therapy duration, h	18.2 ± 60.4	13.2 ± 52.8	21.7 ± 66.2	0.670
Antibiotic therapy duration, days	10.5 ± 3.9	6.8 ± 2.8	13.1 ± 1.9	< 0.001^*^
Probiotic use duration, days	2.3 ± 4.3	0.3 ± 1.0	3.7 ± 5.2	0.014^*^
PPI use duration, days	11.2 ± 3.5	8.3 ± 3.3	13.3 ± 1.7	< 0.001^*^
Prokinetic use duration, days	1.0 ± 3.0	0.3 ± 1.3	1.4 ± 3.7	0.254
Mucosal protectant use duration, days	1.9 ± 3.8	0.8 ± 1.8	2.6 ± 4.6	0.151
Enteral nutrition formula (Peptide-based / Standard / High-calorie / Diabetes-specific / None), n (%)	12 (30.8) / 9 (23.1) / 15 (38.5) / 2 (5.1) / 1 (2.6)	4 (25.0) / 5 (31.3) / 7 (43.8) / 0 (0.0) / 0 (0.0)	8 (34.8) / 4 (17.4) / 8 (34.8) / 2 (8.7) / 1 (4.3)	0.780
Gastrointestinal Symptoms				
Constipation, n (%)	37 (94.9)	16 (100)	21 (91.3)	0.500
Diarrhea, n (%)	9 (23.1)	2 (12.5)	7 (30.4)	0.260
Upper GI bleeding, n (%)	12 (30.8)	4 (25.0)	8 (34.8)	0.730
Abdominal bloating, n (%)	15 (38.5)	5 (31.3)	10 (43.5)	0.520
Gastric retention, n (%)	18 (46.2)	7 (43.8)	11 (47.8)	0.990
Vomiting, n (%)	5 (12.8)	1 (6.3)	4 (17.4)	0.990
ICU Outcomes				
Mechanical ventilation duration, days	4.7 ± 5.0	2.1 ± 3.1	6.6 ± 5.3	0.004^*^
Final airway status (Extubated / Artificial airway in place), n (%)	16 (41.0) /23 (59.0)	12 (75.0) / 4 (25.0)	4 (17.4) /19 (82.6)	< 0.001^*^
ICU mortality, n (%)	2 (5.1)	1 (6.3)	1 (4.3)	0.990

Data are presented as mean ± SD or number (percentage). Variables with skewed distributions are shown as median (IQR). Continuous variables were compared using Student’s t test or the Mann–Whitney U test, as appropriate. Categorical variables were compared using Fisher’s exact test

*Abbreviations*: *LOS* Length of stay, PPI Proton pump inhibitor, *GCS* Glasgow Coma Scale

* *p* < 0.05

### Microbial diversity analysis

#### Alpha diversity

**Fig. 2:**
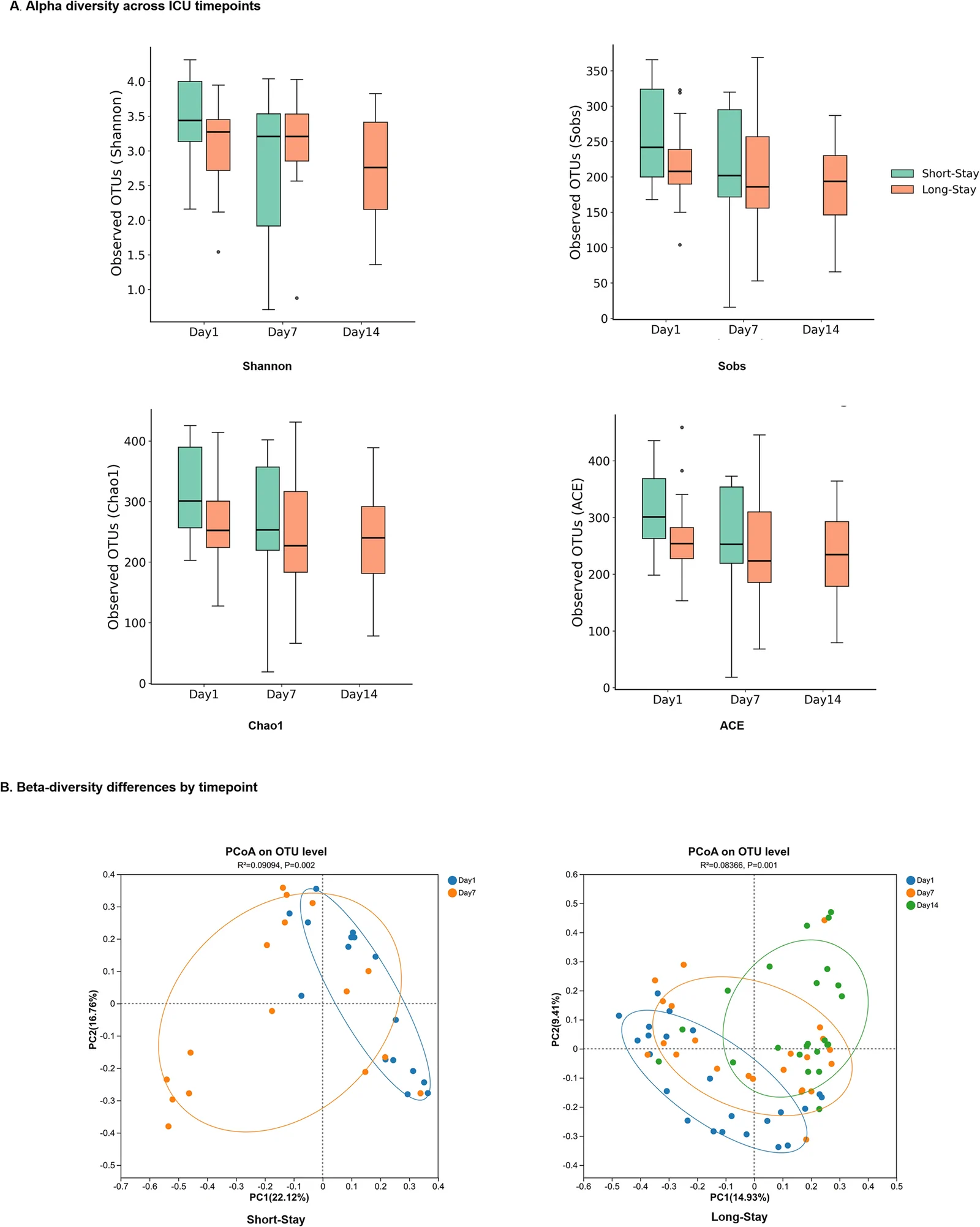
Alpha- and beta-diversity dynamics across ICU timepoints. **A** Changes in alpha diversity indices (Shannon, Sobs, Chao1, and ACE) are shown for both short- and long-stay patients across the three ICU timepoints. All statistical comparisons were adjusted by FDR with a significant threshold of q < 0.05. **B** Principal coordinates analysis (PCoA) based on Bray–Curtis distances illustrates how microbial communities shifted over time within each group. The ellipses represent 95% confidence intervals, and temporal differences were evaluated using PERMANOVA (*p* < 0.05 for significance)

The temporal dynamics of gut microbial alpha diversity were assessed at the OTU level using richness- and diversity-based indices (Sobs, Chao1, ACE, Shannon, Simpson, and Pielou’s evenness). All p-values were adjusted by the FDR method with a statistically significant threshold set at q < 0.05. In patients with short ICU stays (< 14 days), no statistically significant changes were observed between Day 1 and Day 7 after FDR correction. Nevertheless, richness indices (Sobs, Chao1, and ACE) showed a mild downward trend, and the Shannon index showed a similar non-significant pattern, suggesting an early reduction in alpha diversity during initial ICU hospitalization (Fig. 2A; Supplementary Table S1). Among patients with prolonged ICU stays (≥ 14 days), alpha diversity remained statistically stable across Day 1, Day 7, and Day 14, although several indices exhibited a modest decline by Day 7 followed by a slight increase at Day 14 that did not return to baseline levels (all q > 0.05 after FDR correction). This pattern may reflect transient changes in microbial richness and diversity over time (Fig. 2A; Supplementary Table S2). When comparing both groups at matched time points, alpha diversity was broadly comparable at baseline (Day 1) and on Day 7, as shown in Supplementary Table S3 and Supplementary Table S4. The long-stay cohort generally exhibited slightly lower richness and evenness values, but the differences did not reach statistical significance after FDR correction (all q > 0.05). In summary, alpha diversity tended to decline early after ICU admission, particularly in patients with prolonged ICU stays, although these changes were not statistically significant after adjustment for multiple comparisons.

#### Beta diversity

Beta diversity was evaluated using Bray–Curtis dissimilarity and PERMANOVA to assess structural variation in microbial communities over time. In the short-stay group, microbial community composition differed significantly between Day 1 and Day 7 (PERMANOVA *p* = 0.002, R² = 0.09094), indicating significant early temporal restructuring of microbial community composition (Fig. 2B). Among long-stay patients, serial samples across Day 1, Day 7, and Day 14 also showed significant temporal restructuring (*p* = 0.001, R² = 0.08366), with Day 14 exhibiting a separate cluster and greater dispersion, consistent with increasing heterogeneity over time (Fig. 2B). These within-group trends were further reflected in genus-level compositional patterns, as shown in Supplementary Figure S1. Between-group comparisons at matched time points (Day 1 and Day 7) showed no significant differences in overall community structure (PERMANOVA *p* = 0.855 and 0.205, respectively), indicating broadly similar microbial compositions between short- and long-stay patients at baseline and early ICU exposure. The corresponding between-group β-diversity plots are presented in Supplementary Figure S2.

### Differential taxa identification and genus-level validation

**Fig. 3:**
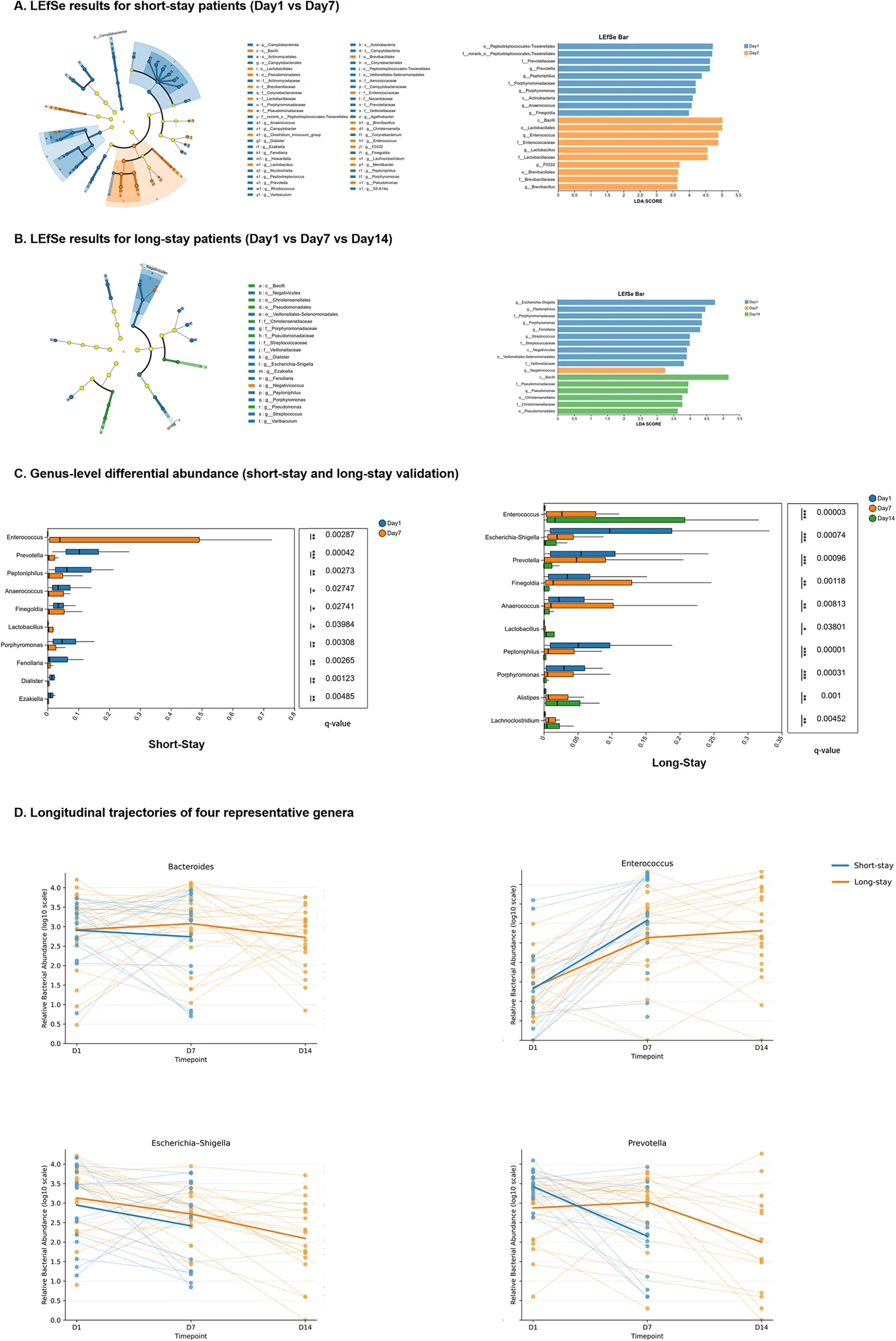
Differential taxa patterns and temporal trajectories in short-stay and long-stay patients. **A** LEfSe-based cladogram and LDA barplot showing differentially abundant taxa between ICU Day 1 and Day 7 in short-stay patients (<14 days). Within-group temporal changes were identified using LEfSe with LDA score > 3.0. **B** LEfSe-based cladogram and LDA barplot showing differentially abundant taxa across ICU Day 1, Day 7, and Day 14 in long-stay patients (≥14 days). Within-group temporal changes were identified using LEfSe with LDA score > 3.0. **C** Genus-level differential abundance validation using FDR-corrected comparisons for short-stay (left) and long-stay (right) patients. Horizontal bar plots show the top 10 genera with the smallest FDR-adjusted q values. Relative abundances are displayed on the x-axis, and corresponding FDR-adjusted q-values are listed on the right side of each panel. **D** Patient-level longitudinal trajectories of four representative genera (*Bacteroides*, *Enterococcus*, *Escherichia–Shigella*, and *Prevotella*) stratified by ICU length of stay. Each point represents log10-transformed relative abundance in an individual sample, and thin lines connect repeated samples from the same patient. Thick lines indicate group-level mean log10-transformed relative abundance for the long-stay and short-stay groups. Values equal to zero were excluded prior to log transformation

LEfSe analysis identified time- and stay-dependent differences in microbial taxa among ICU patients. Genus-level differential testing with FDR correction further validated these patterns. In the short-stay group (< 14 days), gut microbial communities shifted markedly from Day 1 to Day 7. Anaerobic genera such as *Prevotella*, *Veillonella* and *Porphyromonas* decreased substantially, whereas facultative or hospital-adapted genera including *Enterococcus*, *Lactobacillus*, *Brevibacillus* and *Pseudomonas* increased (Fig. 3A; Supplementary Table S5). These findings were confirmed by genus-level statistical validation, which demonstrated significant reductions in anaerobic genera and increases in facultative or opportunistic taxa (FDR-corrected q < 0.05, Fig. 3C, left).

Among long-stay patients (≥ 14 days), microbial restructuring appeared later but became apparent by Day 14. LEfSe identified consistent depletion of *Prevotella* and *Dialister* together with enrichment of *Pseudomonas* and Christensenellaceae-related genera (Fig. 3B; Supplementary Table S5). Additional genera, including *Escherichia–Shigella*, also showed temporal changes consistent with ICU-associated microbial restructuring. Genus-level validation confirmed these patterns and highlighted the gradual emergence of *Enterococcus* as one of the predominant genera by Day 14 (FDR-corrected q < 0.05, Fig. 3C, right).

Between-group comparisons showed broadly similar directional patterns. On Day 1, short-stay patients retained higher abundances of anaerobic genera, including *Prevotella* and *Megasphaera*, whereas long-stay patients showed relatively higher abundances of taxa such as *Synergistes* and Saccharimonadaceae-related genera (Supplementary Figure S3; Supplementary Table S6). By Day 7, long-stay patients demonstrated additional increases in Actinobacteria-related genera, including *Corynebacterium* and *Eggerthella*, while short-stay patients showed relatively higher abundances of genera such as *Lactobacillus* and *Gemella.* Although these between-group differences were less pronounced than the within-group temporal changes, they were directionally consistent with the length-of-stay–related restructuring described above.

### Temporal dynamics of key genera

Because differential-abundance analyses identify taxa that differ at individual timepoints, we next examined longitudinal changes in representative genera from Day 1 to Day 14. Four representative genera related to ICU-associated microbial restructuring were selected: *Bacteroides*, *Enterococcus*, *Escherichia–Shigella* and *Prevotella* (Fig. 3D).

Patient-level trajectories revealed substantial inter-individual variability, but consistent group-level patterns were observed. *Bacteroides*, a common anaerobic gut genus, showed modest changes between Day 1 and Day 7, with heterogeneous individual trajectories; in long-stay patients, a decline was observed by Day 14. *Enterococcus*, a facultative and hospital-adapted genus, increased from Day 1 to Day 7 in both ICU stay groups, with a steeper rise in short-stay patients and sustained elevation through Day 14 in the long-stay group. *Escherichia–Shigella*, a facultative anaerobic genus, decreased from Day 1 to Day 7 in short-stay patients and showed a progressive decline from Day 1 to Day 14 in long-stay patients. *Prevotella*, an obligate anaerobic genus, decreased from Day 1 to Day 7 in both groups, with a further decline by Day 14 in the long-stay group. Overall, these trajectories indicate enrichment of facultative or aerotolerant taxa together with depletion of obligate anaerobes during the ICU course.

### OTU-level stratification and clinical associations

We were unable to identify stable community patterns at the genus level, which may reflect the heterogeneous trajectories of gut communities in ICU patients. Accordingly, unsupervised clustering was performed at the OTU level to derive reproducible OTU-level cluster profiles and to determine whether these profiles corresponded to clinically relevant patient subgroups.

#### OTU-level clustering and subgroup structure

Unsupervised hierarchical clustering identified two distinct subgroups dominated by OTU967 and OTU502, corresponding to the *Escherichia–Shigella* and *Enterococcus* lineages, respectively. Taxonomic annotation based on the SILVA v138 database indicated that OTU967 and OTU502 most closely matched *Escherichia coli* and *Enterococcus faecium*, respectively. Because species-level identification based on 16 S rRNA V3–V4 sequences is inherently uncertain, these assignments are considered tentative, and both OTUs are referred to at the genus level (*Escherichia–Shigella* for OTU967; *Enterococcus* for OTU502) throughout the manuscript.

**Fig. 4:**
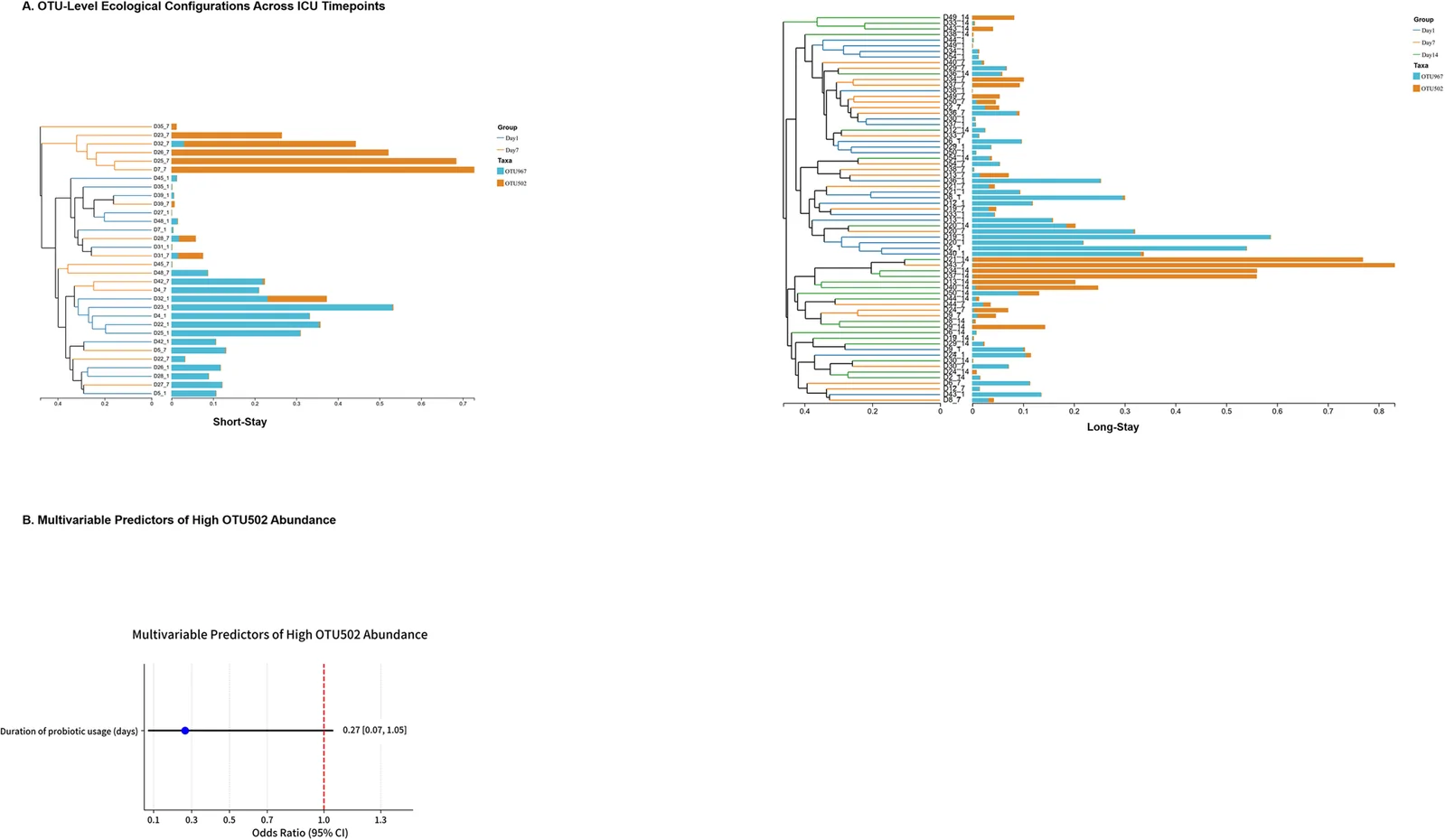
OTU-level dominant profiles and clinical features associated with OTU502 abundance. **A** Hierarchical clustering based on Bray–Curtis dissimilarity displays two distinct OTU-level dominant profiles dominated by either *Enterococcus* (OTU502) or *Escherichia–Shigella* (OTU967). Samples from different timepoints and ICU stay categories are displayed for context. **B** Multivariable logistic regression identifying clinical characteristics associated with high OTU502 abundance; odds ratios are shown with 95% confidence intervals. High and low OTU502 groups were defined using the median relative abundance at ICU Day 7

In the short-stay group (< 14 days), Day 1 and Day 7 samples clustered into distinct branches, indicating a clear change in community structure by Day 7, with increased representation of *Enterococcus*-associated samples (Fig. 4A). In the long-stay group (≥ 14 days), samples were more widely distributed across Day 1, Day 7, and Day 14, with a delayed and heterogeneous emergence of *Enterococcus* by Day 14 (Fig. 4A). Within the short-stay group (< 14 days), patients dominated by OTU502 (*Enterococcus*) showed significantly higher APACHE II scores and lower lowest and discharge GCS scores than those dominated by OTU967 (*Escherichia–Shigella*) (Table 2). Because one patient in the short-stay group died during ICU hospitalization, the subgroup comparison was repeated after excluding this non-survivor, and the significant differences persisted (APACHE II: *p* = 0.003; lowest GCS: *p* = 0.020; discharge GCS: *p* = 0.004). In the long-stay group (≥ 14 days), corresponding comparisons at Day 7 and Day 14 were largely comparable between the two OTU-defined subgroups, although age differed between subgroups at Day 7 (Supplementary Tables S7–S8). Because OTU dominance was determined independently at each timepoint, some patients were classified into different OTU-defined subgroups between Day 7 and Day 14, resulting in the change in subgroup counts observed between Supplementary Tables S7 and S8. After exclusion of the single non-survivor in the long-stay group, the results remained statistically non-significant.

**Table 2:** Clinical characteristics and neurological outcomes of OTU502- and OTU967-dominated subgroups among short-stay ICU patients on day 7

Variable	OTU502 (*n* = 6)	OTU967 (*n* = 10)	*p*-value
Demographics			
Sex (male), n (%)	3 (50.0)	2 (20.0)	0.300
Age, years	56.0 ± 7.5	46.5 ± 12.0	0.104
BMI, kg/m²	24.7 ± 3.3	24.8 ± 4.2	0.974
Hypertension, n (%)	3 (50.0)	1 (10.0)	0.234
Diabetes mellitus, n (%)	0 (0.0)	2 (20.0)	0.500
Clinical Diagnosis and Surgery			
Admitting diagnosis (Supratentorial / Infratentorial / Other), n (%)	1 (16.7) / 3 (50.0) / 2 (33.3)	3 (30.0) / 7 (70.0) / 0 (0.0)	0.304
Operative duration, h	5.8 ± 2.3	6.5 ± 1.8	0.460
Estimated blood loss, mL	400 (175–525)	300 (200–1650)	0.792
Intraoperative hypotension duration, min	0 (0–13.8)	5 (0–15)	0.508
Postoperative complications, n (%)	0 (0.0)	1 (10.0)	> 0.999
Postoperative hypoalbuminemia (Normal / Mild / Moderate / Severe), n (%)	3 (50.0) / 1 (16.7) / 2 (33.3) / 0 (0.0)	6 (60.0) / 3 (30.0) / 0 (0.0) / 1 (10.0)	0.659
Severity and Neurological Scores			
APACHE II score at ICU admission	17.2 ± 3.2	11.9 ± 3.8	0.014^*^
Lowest GCS during ICU stay	8 (5–10)	10 (10–10)	0.041^*^
GCS at discharge	9 (7–13)	15 (14–15)	0.022^*^
Therapies and ICU Exposure			
Vasopressor therapy duration, h	0 (0–52.8)	0 (0–0)	0.375
Antibiotic therapy duration, days	7.5 (5.3–9.8)	5.5 (4.0–8.8)	0.439
Probiotic use duration, days	0 (0–0)	0 (0–0)	> 0.999
PPI use duration, days	7.5 (7–12.5)	7.0 (4.8–11.3)	0.317
Prokinetic use duration, days	0 (0–0)	0 (0–0)	> 0.999
Mucosal protectant use duration, days	0 (0–1.8)	0 (0–2.0)	> 0.999
Enteral nutrition formula (Peptide-based / Standard / High-calorie / Diabetes-specific / None), n (%)	1 (16.7) / 1(16.7) / 4 (66.6) / 0 (0.0) / 0 (0.0)	3 (30.0) / 4 (40.0) / 3 (30.0) / 0 (0.0) / 0 (0.0)	0.313
Gastrointestinal Symptoms			
Constipation, n (%)	6 (100)	10 (100)	> 0.999
Diarrhea, n (%)	2 (33.3)	0 (0.0)	0.125
Upper GI bleeding, n (%)	1 (16.7)	3 (30.0)	> 0.999
Abdominal bloating, n (%)	2 (33.3)	3 (30.0)	> 0.999
Gastric retention, n (%)	3 (50.0)	4 (40.0)	> 0.999
Vomiting, n (%)	1 (16.7)	0 (0.0)	0.375
ICU Outcomes			
Mechanical ventilation duration, days	3 (0–4)	0 (0–2.3)	0.271
Final airway management (Extubated / Artificial airway in place), n (%)	2 (33.3) / 4 (66.7)	2 (20.0) / 8 (80.0)	> 0.999
ICU length of stay, days	9.8 ± 2.2	7.1 ± 2.9	0.070
ICU mortality, n (%)	0 (0.0)	1 (10.0)	> 0.999

Continuous variables are presented as mean ± SD or median (IQR) according to distribution. Categorical variables are reported as number (percentage). Between-group comparisons were performed using the Mann–Whitney U test for continuous variables and Fisher’s exact test for categorical variables. * *p* < 0.05

#### Clinical features associated with OTU502 abundance

Patients were classified into high- and low-OTU502 groups according to the median OTU502 abundance at ICU Day 7. Multivariable logistic regression analysis showed that prolonged probiotic use was associated with a lower likelihood of high OTU502 abundance, although the association did not reach statistical significance (OR = 0.27, 95% CI: 0.07–1.05, *p* = 0.06) (Fig. 4B). No statistically significant associations were observed for other clinical variables, including anaerobic antibiotic exposure defined by presence or absence of anaerobic coverage or by cumulative duration of exposure (all *p* > 0.1).

To account for repeated measurements, linear mixed-effects models (LMMs) were constructed using serial OTU502 abundance across ICU Day 1, Day 7, and Day 14. In this model, longer probiotic exposure showed a negative association with OTU502 abundance (β = −0.043, 95% CI: −0.114 to 0.028, *p* = 0.236), whereas duration of anaerobic antibiotic coverage was not significantly associated with OTU502 abundance (β = −0.035, 95% CI: −0.103 to 0.033, *p* = 0.308).

#### Clinical characteristics in relation to OTU502 dynamics

Analyses of recovery-related clinical measures were restricted to patients who survived to ICU discharge (*n* = 37). Patient-specific OTU502 trajectories were quantified as the slope derived from linear regression of log-transformed OTU502 abundance across ICU Day 1, Day 7, and Day 14. In linear regression analyses, OTU502 slope was not significantly associated with ICU length of stay (β = −6.15, 95% CI: −22.08 to 9.77, *p* = 0.438), GCS score at discharge (β = −0.89, 95% CI: −6.81 to 5.04, *p* = 0.763), or duration of mechanical ventilation (β = 2.90, 95% CI: −5.42 to 11.21, *p* = 0.484). Logistic regression analyses similarly showed no significant associations between OTU502 slope and gastrointestinal complications, including constipation (*p* = 0.574), diarrhea (*p* = 0.323), upper gastrointestinal bleeding (*p* = 0.502), abdominal bloating (*p* = 0.570), gastric retention (*p* = 0.922), vomiting (*p* = 0.919), or final airway management status (*p* = 0.365). Overall, longitudinal changes in OTU502 abundance were not significantly associated with short-term recovery-related clinical measures in this cohort.

### Environmental factors and gut microbiota structure

#### Distance-based redundancy analysis (db-RDA)

**Fig. 5:**
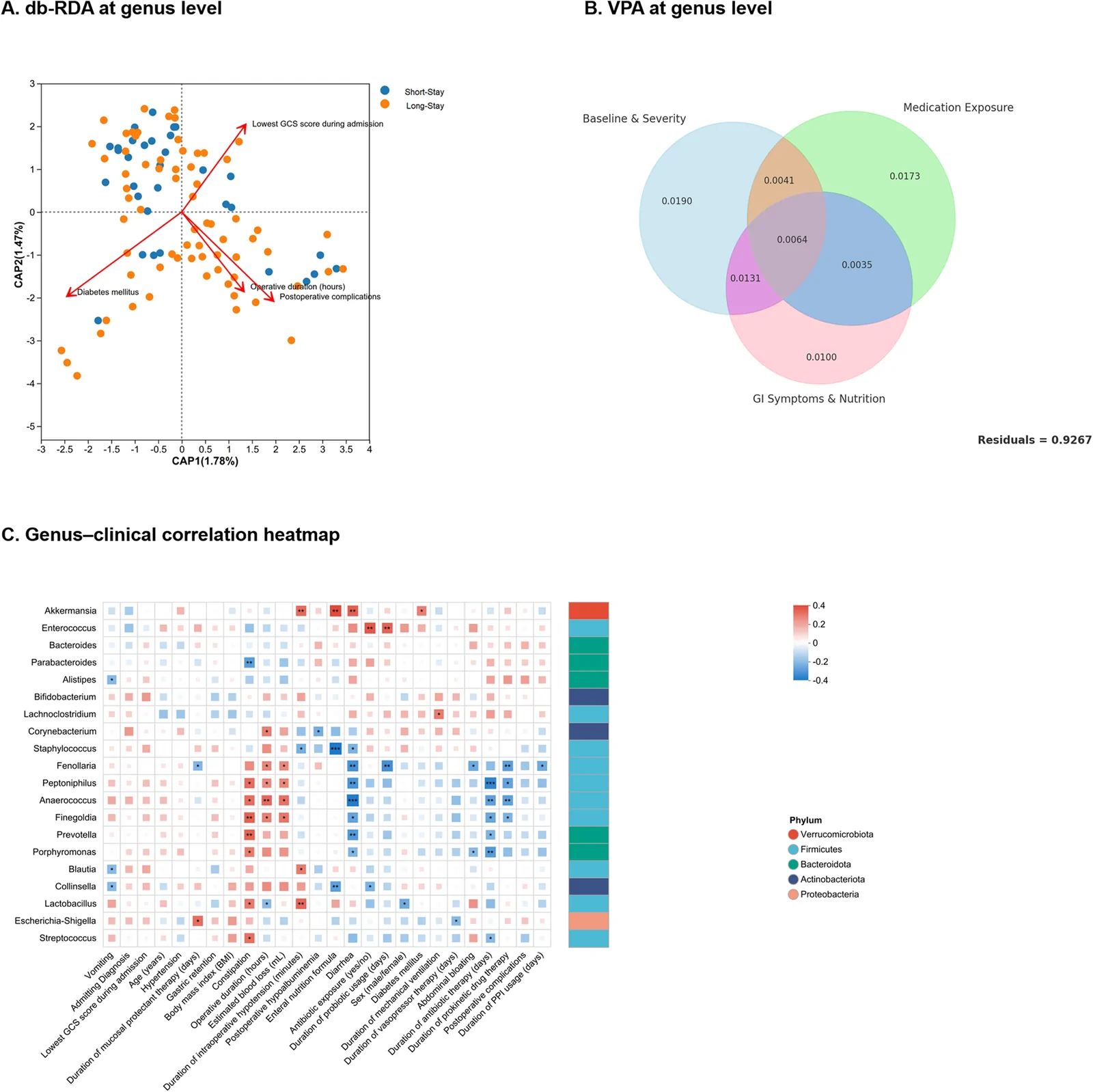
Environmental factors associated with gut microbial community structure. **A** Genus-level db-RDA illustrating the association between key clinical and environmental variables and microbial composition. The first two constrained axes explained 1.78% and 1.47% of the total variance. **B** Variation partitioning analysis (VPA) showing the contributions of baseline and severity indices, medication exposure, as well as gastrointestinal and nutritional factors. Combined, these variables explained 7.33% of the total variance. **C** Genus–clinical correlation heatmap summarizing exploratory associations between dominant genera and key clinical variables. Positive and negative correlations are color-coded, with significance marked by asterisks (*q < 0.05, **q < 0.01, ***q < 0.001)

Distance-based redundancy analysis (db-RDA) was performed at the genus level to evaluate the collective influence of clinical and environmental variables on gut microbial structure (Fig. 5A). To address potential multicollinearity, variance inflation factor (VIF) assessment was conducted prior to model construction. Six variables showed severe collinearity (VIF > 10), including upper gastrointestinal bleeding (UGIB, VIF = 14.69), APACHE II score (VIF = 14.53), mucosal protectant therapy (VIF = 13.52), intraoperative hypotension (VIF = 11.86), duration of antibiotic therapy (VIF = 11.85), and duration of PPI usage (VIF = 11.09). Among these, UGIB and APACHE II score were excluded because of their particularly high collinearity and overlapping clinical implications with other retained variables (e.g., postoperative complications and vasopressor use). The remaining four high-VIF variables were retained due to their established biological and clinical relevance to gut microbiota dynamics and ICU patient management. Among the retained variables, diabetes, operative duration, postoperative complications, and the lowest GCS score showed measurable but modest correlations with overall community composition. The first two constrained axes explained 1.78% and 1.47% of the total variance, respectively, accounting for 3.25% in combination. Although samples from the short- and long-stay groups showed modest separation along these axes, the wide overall variation suggested that clinical and environmental factors had only a limited impact on gut microbial structure.

#### Variation partitioning analysis (VPA)

To quantify the relative contribution of distinct variable categories, variation partitioning analysis (VPA) was conducted using the same set of non-collinear variables (Fig. 5B). Baseline and severity indicators independently accounted for 1.90% of the total variance, followed by pharmacologic exposures (1.73%) and by gastrointestinal and nutritional factors (1.00%). Overlapping contributions among these categories explained additional variance, resulting in a combined explained proportion of 7.33%. The remaining 92.67% of variance was unexplained. At the genus level, correlation analysis demonstrated associations between dominant genera and key clinical variables (Fig. 5C), with FDR-corrected q < 0.05 considered statistically significant.

### Functional prediction of the microbiome

Functional prediction was performed using PICRUSt2 and BugBase. KEGG Level 2 pathways were compared between short-stay and long-stay patients and between OTU502-High and OTU502-Low subgroups (Day 7 abundance). No pathways showed statistical significance after FDR correction (all q > 0.05). BugBase-derived microbial phenotypes were also similar across both comparison sets, with no features reaching statistical significance after FDR correction (all q > 0.05). Related heatmaps and phenotype summaries are provided in Supplementary Figure S4 and Supplementary Table S9.

## Discussion

### Temporal remodeling of the gut microbiota in neurosurgical ICU patients

Longitudinal microbial dynamics in neurosurgical ICU patients remain insufficiently characterized. The serial sampling design used in this study enabled us to describe temporal patterns of gut microbiota restructuring during ICU hospitalization. Previous studies have reported substantial microbiota disruption during critical illness, including early diversity loss, depletion of commensal anaerobes, and enrichment of facultative or pathobiont taxa [3, 20–22]. However, longitudinal trajectories may vary across ICU cohorts. Some studies have emphasized progressive dysbiosis during critical illness [20], whereas Park et al. reported alpha-diversity decline through ICU Day 14 followed by recovery by Day 30 in medical ICU patients admitted with sepsis and receiving broad-spectrum antibiotics [23]. In the present cohort, alpha diversity indices did not show statistically significant differences after multiple-testing correction, although several richness and diversity indices showed a mild decline by Day 7 and a slight increase at Day 14 among long-stay patients. This pattern is broadly consistent with the early decline and later recovery described by Park et al., although our sampling ended at Day 14 and could not assess subsequent recovery.

In parallel, beta diversity analyses demonstrated significant temporal restructuring of microbial community composition. Among long-stay patients, Day 14 samples exhibited greater dispersion of community structure, indicating increased between-patient heterogeneity at later time points. This greater Day 14 dispersion may reflect the combined effects of prolonged ICU course, cumulative treatment exposure, time-at-risk effects, and underlying clinical heterogeneity.

### *Enterococcus* (OTU502) expansion and clinical context

In this cohort, *Enterococcus* (OTU502) expansion was observed in a subset of neurosurgical ICU patients. Higher OTU502 abundance at ICU Day 7 was more frequently observed in patients with greater illness severity and lower GCS scores, and OTU502-dominated microbial profiles were also associated with differences in clinical characteristics in subgroup analyses. These observations indicate an association between OTU502 abundance and clinical status; however, given the observational design of this study, the directionality of this relationship cannot be determined.

These findings are broadly consistent with previous ICU studies in which *Enterococcus* overgrowth has been associated with disrupted microbial communities and adverse clinical status in critically ill patients [23–26]. In the VRE colonisation study by Park et al. [23], VRE-positive samples showed markedly higher sequencing-based *Enterococcus* relative abundance and lower alpha diversity, highlighting *Enterococcus* expansion as a clinically relevant feature of disrupted ICU gut microbial communities.

Although species-level identification is limited by 16S rRNA sequencing, OTU502 was taxonomically annotated as belonging to the *Enterococcus* lineage. Previous studies have reported several biological characteristics of *Enterococcus* species, including tolerance to environmental stress, intrinsic aerotolerance, multidrug resistance, and the ability to persist in disturbed intestinal ecosystems during critical illness [25, 27–29]. These biological features may be relevant to the relative enrichment of *Enterococcus* observed during ICU-associated microbial restructuring. Experimental studies have suggested potential links between enterococcal metabolites and host responses in critical illness [30–32]. However, these mechanisms were not directly investigated in the present study, and such interpretations should be regarded as potential explanations rather than validated pathways in this cohort.

The delayed and heterogeneous emergence of *Enterococcus* in long-stay patients may reflect asynchronous microbial succession rather than a uniform delayed expansion. In short-stay patients, Day 1 and Day 7 samples formed more distinct clusters, suggesting a relatively clear early community transition accompanied by increased representation of *Enterococcus*-associated profiles. By contrast, long-stay patients showed wider sample dispersion across Day 1, Day 7, and Day 14, indicating greater between-patient heterogeneity and less synchronized microbial trajectories. Similar inter-individual variability and pathogen-dominated, low-diversity states have been reported in critically ill patients [20, 21, 23]. Although long-stay patients had longer and more complex ICU-related exposures, no single treatment factor consistently explained OTU502 abundance in this cohort. Thus, the later emergence of *Enterococcus* may reflect heterogeneous and cumulative ecological pressures during prolonged ICU care rather than a single identifiable mechanism. This interpretation remains speculative.

### Neuro–ICU–gut interactions in the context of microbial restructuring

These findings suggest that neurological injury, ICU-related exposures, and gut microbial restructuring may be interconnected in neurosurgical critical illness. Based on the patterns observed in this cohort, these relationships can be interpreted within a conceptual framework linking neurocritical illness, ICU care, and gut microbial dynamics.

In neurosurgical patients, brain injury and postoperative stress responses may influence gastrointestinal physiology through altered autonomic regulation, systemic responses, impaired intestinal function, and changes in mucosal perfusion [33]. These mechanisms provide a biologically plausible context for gut microbial alterations in neurocritical illness, although they were not directly assessed in the present study.

At the same time, treatment-related ICU exposures may also be relevant factors in the observed microbial restructuring and should be considered when interpreting these findings [20, 24, 34–36]. In this cohort, patients with prolonged ICU stays had significantly longer exposure to both antibiotic therapy and proton pump inhibitor (PPI) therapy than short-stay patients. Antibiotic exposure, particularly regimens with anti-anaerobic activity, has been shown to reduce gut microbial density and disrupt commensal anaerobic communities in critically ill or hospitalized patients [37, 38]. These effects are consistent with the depletion of anaerobic genera observed in the present cohort. PPI therapy has also been associated with altered gut microbial ecology, potentially through reduced gastric acid barrier function and changes in upper gastrointestinal microbial translocation [39]. These observations provide relevant context for interpreting the stay-dependent microbial restructuring observed in this cohort. However, because these treatment exposures were closely linked to ICU length of stay and overall clinical course, their independent effects could not be disentangled in the present study.

### Environmental influences and clinical implications

Our multivariate analyses showed that measured ICU exposures accounted for only 3–7% of community variance, indicating that the clinical variables examined explained only a limited proportion of the observed microbial variability. Even under broadly similar treatments, patients demonstrated distinct microbial patterns. These findings suggest that host-related clinical features may represent one measurable contributor within the variables examined, while most variation likely reflects additional unmeasured factors [21, 40].

The Day 7 microbial profiles may represent a transitional phase in which community structures became more differentiated. This time point may represent a useful window for monitoring early microbial restructuring during ICU hospitalization. Evaluating microbiota changes at this stage, together with clinical parameters such as GCS trends and antibiotic exposure, may provide a reference for future studies of microbial restructuring during ICU hospitalization.

From a translational perspective, these findings suggest that future studies should evaluate factors associated with microbial balance rather than focusing only on individual ICU exposures. Factors related to microbial balance, including preservation of anaerobic communities and conditions associated with expansion of facultative taxa, may be more informative than any single exposure when interpreting ICU-associated microbial restructuring. In clinical practice, this may include consideration of antibiotic and acid-suppressive therapy [35, 41], early nutritional support, and further evaluation of microbiota-targeted interventions in controlled studies. A non-significant trend toward lower OTU502 abundance with longer probiotic exposure was observed in this cohort. Given the imbalance in probiotic exposure duration between groups, a potential contribution of probiotic administration to specific taxa such as *Lactobacillus* cannot be excluded.

In neurosurgical ICU patients, microbiota-targeted approaches may also need to consider neurological influences on gastrointestinal physiology. Future studies integrating microbiome profiling with metabolomic or physiological measurements may help clarify the relationship between microbial restructuring and clinical status in this population [22, 42].

In addition, functional prediction analyses based on PICRUSt2 did not identify statistically significant differences in KEGG pathways between comparison groups after FDR correction. This result may reflect the limited functional resolution of 16S rRNA–based prediction methods, as well as the modest sample size of the present cohort. Alternatively, the observed microbial alterations may primarily represent shifts in community composition rather than large-scale differences in predicted metabolic functions. Further studies using metagenomic or metabolomic approaches will be required to clarify the functional consequences of these microbial changes.

### Limitations

The study has several limitations. Its single-center design and modest sample size may limit generalizability. Another limitation is the absence of non-neurosurgical ICU or non-ICU neurosurgical comparator groups, limiting our ability to separate neurological injury-related microbial features from changes associated with critical illness and ICU-related exposures. The modest cohort size also limits the interpretation of associations between OTU502 and clinical characteristics. In addition, 16S rRNA sequencing provides limited functional and strain-level resolution. Multi-center cohorts with appropriate comparator groups and metagenomic or metabolomic profiling are needed to better characterize gut microbial dynamics and clarify the biological significance of OTU502.

## Conclusions

Neurosurgical ICU patients in this cohort exhibited longitudinal gut microbiome trajectories characterized by early community disruption, partial later recovery, and *Enterococcus* (OTU502) expansion. OTU502 enrichment was observed as a microbial feature associated with differences in clinical status within this cohort, but its clinical significance remains to be clarified. These findings provide a descriptive framework for understanding gut microbiome dynamics during neurosurgical ICU care and support further investigation in larger, appropriately controlled studies.

## Supplementary Information

Additional file 1. Supplementary Figure S1.

Additional file 2. Supplementary Figure S2.

Additional file 3. Supplementary Figure S3.

Additional file 4. Supplementary Figure S4.

Additional file 5. Supplementary Tables S1–S9.

## Data Availability

The raw sequencing data generated in this study have been deposited in the NCBI Sequence Read Archive (SRA) under BioProject accession number PRJNA1461393.

## Abbreviations

APACHE II: Acute Physiology and Chronic Health Evaluation II
CI: Confidence interval
db-RDA: Distance based redundancy analysis
DNA: Deoxyribonucleic acid
FDR: False discovery rate
GCS: Glasgow Coma Scale
GI: Gastrointestinal
KEGG: Kyoto Encyclopedia of Genes and Genomes
LDA: Linear discriminant analysis
LEfSe: Linear discriminant analysis effect size
LOS: Length of stay
NLRP3: NOD-like receptor protein 3
OR: Odds ratio
OTU: Operational taxonomic unit
PCoA: Principal coordinates analysis
PICRUSt2: Phylogenetic Investigation of Communities by Reconstruction of Unobserved States 2
PPI: Proton pump inhibitor
rRNA: Ribosomal RNA
UGIB: Upper gastrointestinal bleeding
VIF: Variance inflation factor
VPA: Variation partitioning analysis
