# Supplementary material for: Longitudinal gut microbiome dynamics in neurosurgical ICU patients: Enterococcus expansion as a feature of microbial restructuring
Source: BMC Microbiol. 2026 Aug 17;26:1000. doi: 10.1186/s12866-026-05450-5 (PMC13757225; doi:10.1186/s12866-026-05450-5)

A. Short-stay group

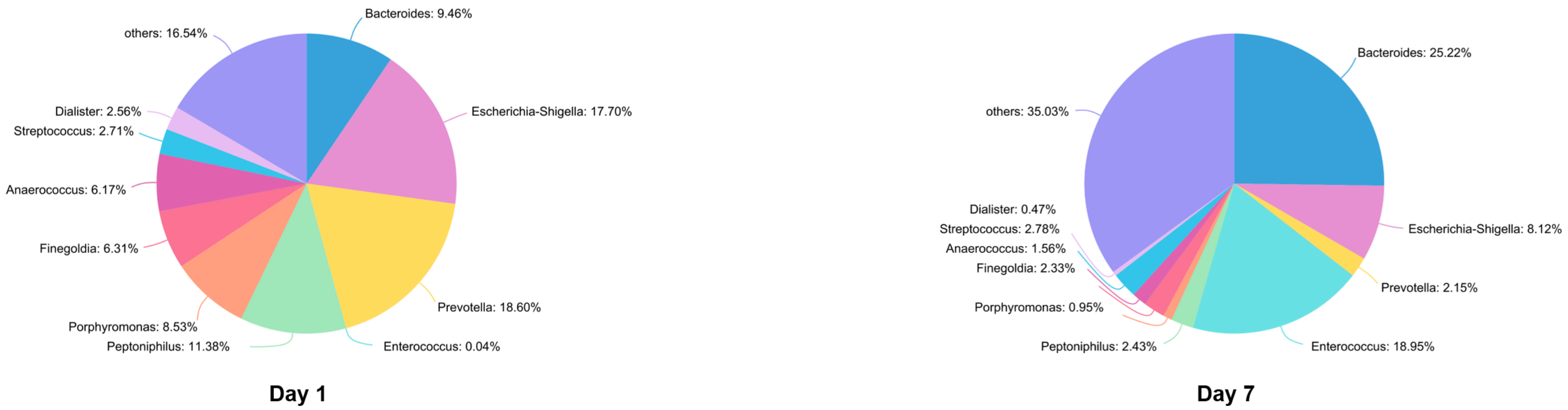

B. Long-stay group

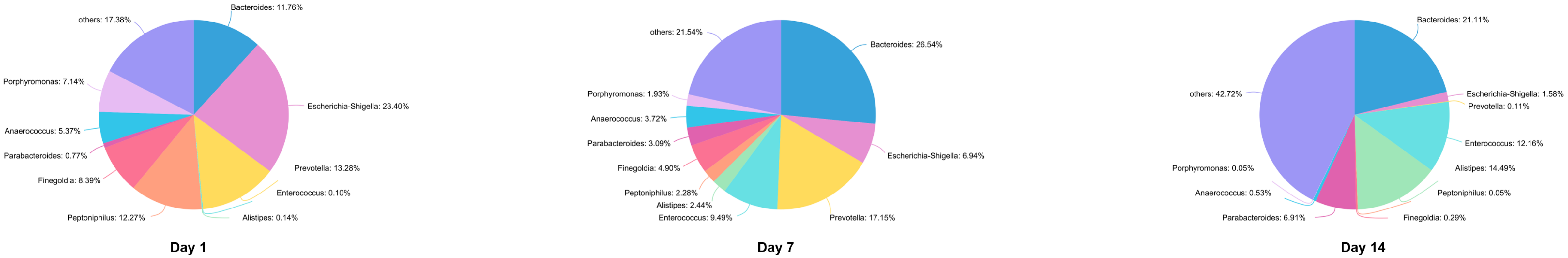

Supplement: Supplementary file 1 [file 12866_2026_5450_MOESM1_ESM.pdf]
