# Supplementary material for: Longitudinal gut microbiome dynamics in neurosurgical ICU patients: Enterococcus expansion as a feature of microbial restructuring
Source: BMC Microbiol. 2026 Aug 17;26:1000. doi: 10.1186/s12866-026-05450-5 (PMC13757225; doi:10.1186/s12866-026-05450-5)
Supplement: Supplementary file 2 [file 12866_2026_5450_MOESM2_ESM.pdf]

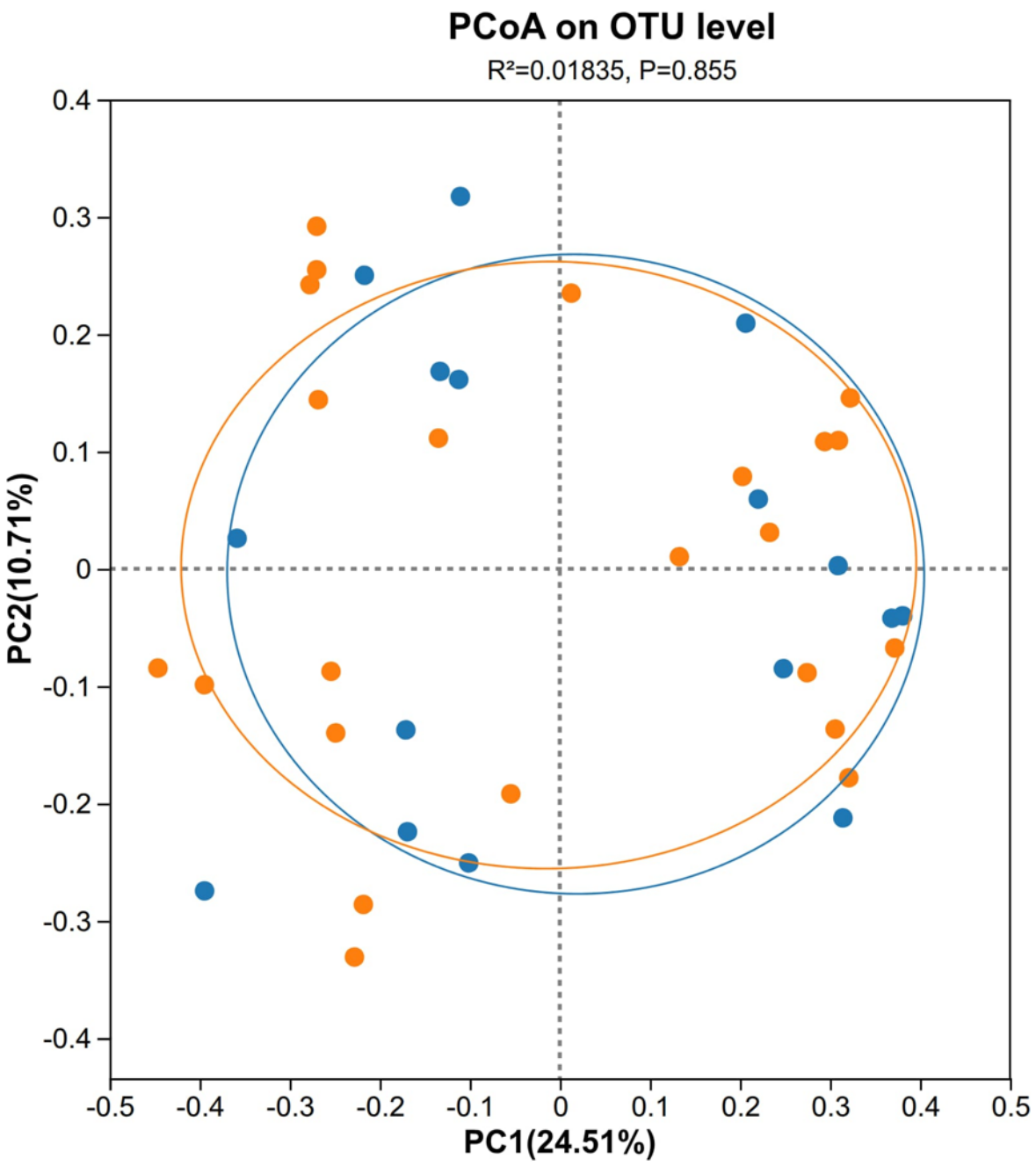
PCoA on OTU level
R²=0.01835, P=0.855
PC1(24.51%)
PC2(10.71%)
-0.5
-0.4
-0.3
-0.2
-0.1
0
0.1
0.2
0.3
0.4
0.5
0.4
0.3
0.2
0.1
0
-0.1
-0.2
-0.3
-0.4

Day 1

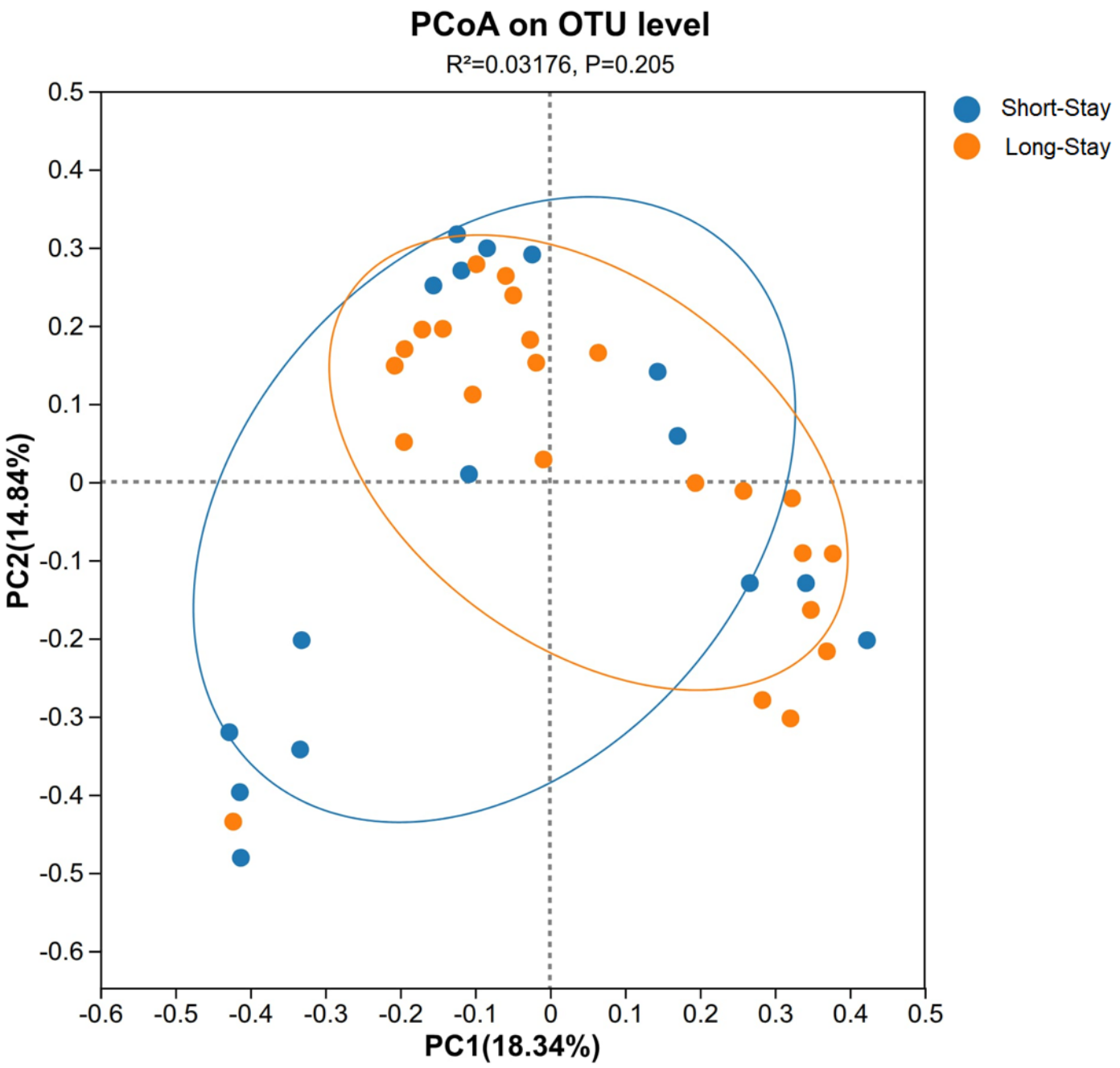
PCoA on OTU level
R²=0.03176, P=0.205
Short-Stay
Long-Stay
PC1(18.34%)
PC2(14.84%)
-0.6
-0.5
-0.4
-0.3
-0.2
-0.1
0
0.1
0.2
0.3
0.4
0.5
0.5
0.4
0.3
0.2
0.1
0
-0.1
-0.2
-0.3
-0.4
-0.5
-0.6

Day 7
