# Supplementary material for: Longitudinal gut microbiome dynamics in neurosurgical ICU patients: Enterococcus expansion as a feature of microbial restructuring
Source: BMC Microbiol. 2026 Aug 17;26:1000. doi: 10.1186/s12866-026-05450-5 (PMC13757225; doi:10.1186/s12866-026-05450-5)
Supplement: Supplementary file 5 [file 12866_2026_5450_MOESM5_ESM.docx]

**Supplementary Tables**

**Table S1. Comparison of Alpha Diversity Indices Between Day 1 and Day 7 in the Short-Stay Group (<14 days, n = 16)**

| **Index** | **Day 1 (mean ± SD)** | **Day 7 (mean ± SD)** | **p-value** | **FDR-adjusted p-value** |
| --- | --- | --- | --- | --- |
| **Sobs** | 253.75 ± 66.84 | 212.12 ± 87.14 | 0.15 | 0.18 |
| **Shannon** | 3.45 ± 0.57 | 2.84 ± 1.01 | 0.08 | 0.18 |
| **Simpson** | 0.09 ± 0.07 | 0.18 ± 0.18 | 0.16 | 0.18 |
| **ACE** | 310.79 ± 66.68 | 259.07 ± 102.61 | 0.13 | 0.18 |
| **Chao1** | 314.50 ± 74.04 | 262.20 ± 108.27 | 0.14 | 0.18 |
| **Coverage** | 1.00 ± 0.00 | 1.00 ± 0.00 | 0.56 | 0.56 |
| **Pielou** | 0.63 ± 0.08 | 0.53 ± 0.15 | 0.08 | 0.18 |

Values are presented as mean ± standard deviation. P-values were calculated using the Wilcoxon signed-rank test and corrected for multiple comparisons using the false discovery rate (FDR).

**Table S2. Comparison of Alpha Diversity Indices Across Day 1, Day 7, and Day 14 in the Long-Stay Group (≥14 days, n = 23)**

| **Index** | **Day 1**  **(mean ± SD)** | **Day 7**  **(mean ± SD)** | **Day 14**  **(mean ± SD)** | **p-value (Day1 vs. 14)** | **p-value (Day7 vs. 1)** | **p-value (Day14 vs. 7)** | **Overall p-value** | **FDR-adjusted p-value** |
| --- | --- | --- | --- | --- | --- | --- | --- | --- |
| **Sobs** | 217.35± 52.79 | 185.48± 60.47 | 202.48± 74.76 | 0.36 | 0.66 | 0.66 | 0.29 | 0.37 |
| **Shannon** | 3.08 ± 0.60 | 2.75 ± 0.78 | 3.12 ± 0.64 | 0.37 | 0.74 | 0.29 | 0.21 | 0.37 |
| **Simpson** | 0.13 ± 0.10 | 0.19 ± 0.14 | 0.12 ± 0.13 | 0.28 | 0.83 | 0.27 | 0.18 | 0.37 |
| **ACE** | 268.58 ± 63.72 | 229.40 ± 76.25 | 245.18 ± 88.04 | 0.39 | 0.52 | 0.70 | 0.29 | 0.37 |
| **Chao1** | 265.44 ± 64.99 | 231.27 ± 82.38 | 244.13 ± 88.52 | 0.49 | 0.54 | 0.77 | 0.34 | 0.37 |
| **Coverage** | 1.00 ± 0.00 | 1.00 ± 0.00 | 1.00 ± 0.00 | 0.61 | 0.61 | 0.92 | 0.37 | 0.37 |
| **Pielou** | 0.57 ± 0.10 | 0.53 ± 0.13 | 0.59 ± 0.10 | 0.42 | 0.56 | 0.20 | 0.17 | 0.37 |

Values are presented as mean ± standard deviation. The Kruskal–Wallis test evaluated differences across timepoints, followed by Dunn’s post-hoc test with FDR correction.

**Table S3.** **Comparison of Alpha Diversity Indices Between ICU Length of Stay (<14 Days vs. ≥14 Days) on Day 1**

| **Index** | **ICU LOS < 14 days**  **(mean ± SD)** | **ICU LOS ≥ 14 days**  **(mean ± SD)** | **p-value** | **FDR-adjusted p-value** |
| --- | --- | --- | --- | --- |
| **Sobs** | 253.75 ± 66.84 | 217.35 ± 52.78 | 0.0798 | 0.1026 |
| **Shannon** | 3.45 ± 0.57 | 3.08 ± 0.60 | 0.0560 | 0.1026 |
| **Simpson** | 0.09 ± 0.07 | 0.13 ± 0.10 | 0.1197 | 0.1197 |
| **ACE** | 310.79 ± 66.68 | 268.58 ± 63.72 | 0.0565 | 0.1026 |
| **Chao1** | 314.50 ± 74.04 | 265.44 ± 64.99 | 0.0408 | 0.1026 |
| **Coverage** | 1.00 ± 0.00 | 1.00 ± 0.00 | 0.0834 | 0.1026 |
| **Pielou** | 0.63 ± 0.08 | 0.57 ± 0.10 | 0.0879 | 0.1026 |

Values are presented as mean ± standard deviation. Between-group comparisons were performed using the Mann–Whitney U test and corrected for multiple comparisons using the false discovery rate (FDR).

| **Index** | **ICU LOS < 14 days**  **(mean ± SD)** | **ICU LOS ≥ 14 days**  **(mean ± SD)** | **p-value** | **FDR-adjusted p-value** |
| --- | --- | --- | --- | --- |
| **Sobs** | 212.12 ± 87.14 | 202.48 ± 74.76 | 0.7214 | 0.7214 |
| **Shannon** | 2.84 ± 1.01 | 3.12 ± 0.64 | 0.3389 | 0.7214 |
| **Simpson** | 0.18 ± 0.18 | 0.12 ± 0.13 | 0.5393 | 0.7214 |
| **ACE** | 259.07 ± 102.61 | 245.18 ± 88.04 | 0.6629 | 0.7214 |
| **Chao1** | 262.20 ± 108.27 | 244.13 ± 88.52 | 0.5856 | 0.7214 |
| **Coverage** | 1.00 ± 0.00 | 1.00 ± 0.00 | 0.4871 | 0.7214 |
| **Pielou** | 0.53 ± 0.15 | 0.59 ± 0.10 | 0.4493 | 0.7214 |

**Table S4. Comparison of Alpha Diversity Indices Between ICU Length of Stay (<14 Days vs. ≥14 Days) on Day 7**

Values are presented as mean ± standard deviation. Between-group comparisons were performed using the Mann–Whitney U test and corrected for multiple comparisons using the false discovery rate (FDR).

**Table S5**. **Differentially enriched taxa identified by LEfSe within ICU length-of-stay groups. Taxa were selected using thresholds of LDA > 3.0 and p < 0.05.**

| **Comparison type** | **ICU group (s)** | **Time point**  **(s)** | **Phylum** | **Order** | **Family** | **Genus** | **LDA score** | **p-value** | **Mean abundance** | **Enriched in** | |
| --- | --- | --- | --- | --- | --- | --- | --- | --- | --- | --- | --- |
| Within-group | Short-stay | Day 1 vs Day 7 | Firmicutes | Peptostreptococcales-Tissierellales | Peptostreptococcaceae | *Peptostreptococcus* | 3.52 | 0.02 | 3.82 | Day 1 ↑ |  |
| Within-group | Short-stay | Day 1 vs Day 7 | Firmicutes | Peptostreptococcales-Tissierellales | norank_o__Peptostreptococcales-Tissierellales | *Murdochiella* | 3.54 | 0.01 | 4.05 | Day 1 ↑ |  |
| Within-group | Short-stay | Day 1 vs Day 7 | Firmicutes | Peptostreptococcales-Tissierellales | norank_o__Peptostreptococcales-Tissierellales | Unclassified | 4.70 | 0.01 | 5.41 | Day 1 ↑ |  |
| Within-group | Short-stay | Day 1 vs Day 7 | Firmicutes | Brevibacillales | Brevibacillaceae | *Brevibacillus* | 3.64 | 0.04 | 1.07 | Day 7 ↑ |  |
| Within-group | Short-stay | Day 1 vs Day 7 | Firmicutes | Peptostreptococcales-Tissierellales | Unclassified | Unclassified | 4.72 | 0.01 | 5.43 | Day 1 ↑ |  |
| Within-group | Short-stay | Day 1 vs Day 7 | Firmicutes | Veillonellales-Selenomonadales | Unclassified | Unclassified | 3.64 | 0.02 | 4.36 | Day 1 ↑ |  |
| Within-group | Short-stay | Day 1 vs Day 7 | Firmicutes | Veillonellales-Selenomonadales | Veillonellaceae | *Dialister* | 3.65 | 0.00 | 4.19 | Day 1 ↑ |  |
| Within-group | Short-stay | Day 1 vs Day 7 | Actinobacteriota |  | Unclassified | Unclassified | 4.11 | 0.03 | 4.70 | Day 1 ↑ |  |
| Within-group | Short-stay | Day 1 vs Day 7 | Proteobacteria | Pseudomonadales | Pseudomonadaceae | Unclassified | 3.55 | 0.02 | 1.47 | Day 7 ↑ |  |
| Within-group | Short-stay | Day 1 vs Day 7 | Actinobacteriota | Corynebacteriales | Corynebacteriaceae | Unclassified | 3.49 | 0.03 | 4.08 | Day 1 ↑ |  |
| Within-group | Short-stay | Day 1 vs Day 7 | Firmicutes | Peptostreptococcales-Tissierellales | norank_o__Peptostreptococcales-Tissierellales | *Anaerococcus* | 4.08 | 0.03 | 4.75 | Day 1 ↑ |  |
| Within-group | Short-stay | Day 1 vs Day 7 | Actinobacteriota | Corynebacteriales | Corynebacteriaceae | *Corynebacterium* | 3.51 | 0.01 | 3.97 | Day 1 ↑ |  |
| Within-group | Short-stay | Day 1 vs Day 7 | Firmicutes | Peptostreptococcales-Tissierellales | norank_o__Peptostreptococcales-Tissierellales | *Finegoldia* | 3.99 | 0.03 | 4.74 | Day 1 ↑ |  |
| Within-group | Short-stay | Day 1 vs Day 7 | Firmicutes | Brevibacillales | Unclassified | Unclassified | 3.66 | 0.04 | 1.07 | Day 7 ↑ |  |
| Within-group | Short-stay | Day 1 vs Day 7 | Actinobacteriota | Corynebacteriales | Unclassified | Unclassified | 3.61 | 0.00 | 4.17 | Day 1 ↑ |  |
| Within-group | Short-stay | Day 1 vs Day 7 | Campilobacterota | Campylobacterales | Unclassified | Unclassified | 3.50 | 0.00 | 3.99 | Day 1 ↑ |  |
| Within-group | Short-stay | Day 1 vs Day 7 | Firmicutes | Erysipelotrichales | Erysipelotrichaceae | *Merdibacter* | 3.45 | 0.04 | 1.45 | Day 7 ↑ |  |
| Within-group | Short-stay | Day 1 vs Day 7 | Firmicutes | Christensenellales | Christensenellaceae | *Christensenella* | 3.31 | 0.02 | 2.04 | Day 7 ↑ |  |
| Within-group | Short-stay | Day 1 vs Day 7 | Firmicutes | Lachnospirales | Lachnospiraceae | *Lachnoclostridium* | 3.34 | 0.01 | 3.71 | Day 7 ↑ |  |
| Within-group | Short-stay | Day 1 vs Day 7 | Firmicutes | Lactobacillales | Unclassified | Unclassified | 5.01 | 0.02 | 5.48 | Day 7 ↑ |  |
| Within-group | Short-stay | Day 1 vs Day 7 | Proteobacteria | Pseudomonadales | Pseudomonadaceae | *Pseudomonas* | 3.56 | 0.02 | 1.47 | Day 7 ↑ |  |
| Within-group | Short-stay | Day 1 vs Day 7 | Firmicutes | Peptostreptococcales-Tissierellales | norank_o__Peptostreptococcales-Tissierellales | *Ezakiella* | 3.62 | 0.00 | 4.16 | Day 1 ↑ |  |
| Within-group | Short-stay | Day 1 vs Day 7 | Firmicutes | Lactobacillales | Enterococcaceae | *Enterococcus* | 4.89 | 0.00 | 5.31 | Day 7 ↑ |  |
| Within-group | Short-stay | Day 1 vs Day 7 | Firmicutes | Lachnospirales | Lachnospiraceae | *Howardella* | 3.22 | 0.01 | 2.04 | Day 1 ↑ |  |
| Within-group | Short-stay | Day 1 vs Day 7 | Firmicutes | Veillonellales-Selenomonadales | Veillonellaceae | Unclassified | 3.63 | 0.02 | 4.35 | Day 1 ↑ |  |
| Within-group | Short-stay | Day 1 vs Day 7 | Bacteroidota | Bacteroidales | Prevotellaceae | Unclassified | 4.64 | 0.00 | 5.12 | Day 1 ↑ |  |
| Within-group | Short-stay | Day 1 vs Day 7 | Bacteroidota | Bacteroidales | Prevotellaceae | *Prevotella* | 4.64 | 0.00 | 5.12 | Day 1 ↑ |  |
| Within-group | Short-stay | Day 1 vs Day 7 | Proteobacteria | Pseudomonadales | Unclassified | Unclassified | 3.53 | 0.01 | 1.56 | Day 7 ↑ |  |
| Within-group | Short-stay | Day 1 vs Day 7 | Firmicutes | Lactobacillales | Aerococcaceae | Unclassified | 3.46 | 0.03 | 3.71 | Day 1 ↑ |  |
| Within-group | Short-stay | Day 1 vs Day 7 | Actinobacteriota | Actinomycetales | Actinomycetaceae | *Varibaculum* | 3.31 | 0.00 | 3.63 | Day 1 ↑ |  |
| Within-group | Short-stay | Day 1 vs Day 7 | Bacteroidota | Bacteroidales | Porphyromonadaceae | Unclassified | 4.20 | 0.00 | 4.77 | Day 1 ↑ |  |
| Within-group | Short-stay | Day 1 vs Day 7 | Firmicutes | Lactobacillales | Lactobacillaceae | *Lactobacillus* | 4.56 | 0.03 | 4.90 | Day 7 ↑ |  |
| Within-group | Short-stay | Day 1 vs Day 7 | Bacteroidota | Bacteroidales | Porphyromonadaceae | *Porphyromonas* | 4.20 | 0.00 | 4.77 | Day 1 ↑ |  |
| Within-group | Short-stay | Day 1 vs Day 7 | Campilobacterota | Unclassified | Unclassified | Unclassified | 3.50 | 0.00 | 3.99 | Day 1 ↑ |  |
| Within-group | Short-stay | Day 1 vs Day 7 | Actinobacteriota | Actinomycetales | Actinomycetaceae | Unclassified | 3.65 | 0.00 | 4.11 | Day 1 ↑ |  |
| Within-group | Short-stay | Day 1 vs Day 7 | Firmicutes | Lactobacillales | Lactobacillaceae | Unclassified | 4.56 | 0.03 | 4.90 | Day 7 ↑ |  |
| Within-group | Short-stay | Day 1 vs Day 7 | Firmicutes | Brevibacillales | Brevibacillaceae | Unclassified | 3.65 | 0.04 | 1.07 | Day 7 ↑ |  |
| Within-group | Short-stay | Day 1 vs Day 7 | Actinobacteriota | Actinomycetales | Actinomycetaceae | *F0332* | 3.71 | 0.04 | 1.07 | Day 7 ↑ |  |
| Within-group | Short-stay | Day 1 vs Day 7 | Firmicutes | Lactobacillales | Enterococcaceae | Unclassified | 4.89 | 0.00 | 5.31 | Day 7 ↑ |  |
| Within-group | Short-stay | Day 1 vs Day 7 | Campilobacterota | Campylobacterales | Campylobacteraceae | Unclassified | 3.50 | 0.00 | 3.99 | Day 1 ↑ |  |
| Within-group | Short-stay | Day 1 vs Day 7 | Firmicutes | Peptostreptococcales-Tissierellales | Anaerovoracaceae | *S5-A14a* | 3.06 | 0.03 | 3.59 | Day 1 ↑ |  |
| Within-group | Short-stay | Day 1 vs Day 7 | Actinobacteriota | Corynebacteriales | Nocardiaceae | Unclassified | 3.09 | 0.00 | 3.45 | Day 1 ↑ |  |
| Within-group | Short-stay | Day 1 vs Day 7 | Firmicutes | Peptostreptococcales-Tissierellales | norank_o__Peptostreptococcales-Tissierellales | *Fenollaria* | 3.96 | 0.00 | 4.49 | Day 1 ↑ |  |
| Within-group | Short-stay | Day 1 vs Day 7 | Actinobacteriota | Corynebacteriales | Nocardiaceae | *Rhodococcus* | 3.09 | 0.00 | 3.45 | Day 1 ↑ |  |
| Within-group | Short-stay | Day 1 vs Day 7 | Actinobacteriota | Actinomycetales | Unclassified | Unclassified | 3.65 | 0.00 | 4.11 | Day 1 ↑ |  |
| Within-group | Short-stay | Day 1 vs Day 7 | Campilobacterota |  | Unclassified | Unclassified | 3.50 | 0.00 | 3.99 | Day 1 ↑ |  |
| Within-group | Short-stay | Day 1 vs Day 7 | Campilobacterota | Campylobacterales | Campylobacteraceae | *Campylobacter* | 3.50 | 0.00 | 3.99 | Day 1 ↑ |  |
| Within-group | Short-stay | Day 1 vs Day 7 | Firmicutes | Erysipelotrichales | Erysipelotrichaceae | *Clostridium_innocuum_group* | 3.19 | 0.02 | 3.62 | Day 7 ↑ |  |
| Within-group | Short-stay | Day 1 vs Day 7 | Firmicutes | Lachnospirales | Lachnospiraceae | *Agathobacter* | 3.24 | 0.05 | 3.62 | Day 1 ↑ |  |
| Within-group | Short-stay | Day 1 vs Day 7 | Firmicutes | Peptostreptococcales-Tissierellales | norank_o__Peptostreptococcales-Tissierellales | *Peptoniphilus* | 4.38 | 0.00 | 4.92 | Day 1 ↑ |  |
| Within-group | Short-stay | Day 1 vs Day 7 | Firmicutes | Unclassified | Unclassified | Unclassified | 5.01 | 0.01 | 5.49 | Day 7 ↑ |  |
| Within-group | Long-stay | Day 1 vs Day 14 | Firmicutes | Unclassified | Unclassified | Unclassified | 3.91 | 0.00 | 4.41 | Day 1 ↑ |  |
| Within-group | Long-stay | Day 1 vs Day 14 | Firmicutes | Veillonellales-Selenomonadales | Unclassified | Unclassified | 3.90 | 0.00 | 4.30 | Day 1 ↑ |  |
| Within-group | Long-stay | Day 1 vs Day 14 | Firmicutes | Veillonellales-Selenomonadales | Veillonellaceae | *Dialister* | 3.68 | 0.00 | 4.08 | Day 1 ↑ |  |
| Within-group | Long-stay | Day 1 vs Day 14 | Proteobacteria | Pseudomonadales | Pseudomonadaceae | Unclassified | 3.95 | 0.00 | 3.74 | Day 1 ↑ |  |
| Within-group | Long-stay | Day 1 vs Day 14 | Firmicutes | Christensenellales | Unclassified | Unclassified | 3.76 | 0.00 | 4.20 | Day 1 ↑ |  |
| Within-group | Long-stay | Day 1 vs Day 14 | Proteobacteria | Pseudomonadales | Pseudomonadaceae | *Pseudomonas* | 3.93 | 0.00 | 3.74 | Day 1 ↑ |  |
| Within-group | Long-stay | Day 1 vs Day 14 | Firmicutes | Peptostreptococcales-Tissierellales | norank_o__Peptostreptococcales-Tissierellales | *Ezakiella* | 3.63 | 0.00 | 3.96 | Day 1 ↑ |  |
| Within-group | Long-stay | Day 1 vs Day 14 | Firmicutes | Veillonellales-Selenomonadales | Veillonellaceae | Unclassified | 3.81 | 0.00 | 4.22 | Day 1 ↑ |  |
| Within-group | Long-stay | Day 1 vs Day 14 | Proteobacteria | Pseudomonadales | Unclassified | Unclassified | 3.63 | 0.00 | 3.74 | Day 1 ↑ |  |
| Within-group | Long-stay | Day 1 vs Day 14 | Actinobacteriota | Actinomycetales | Actinomycetaceae | *Varibaculum* | 3.24 | 0.00 | 3.60 | Day 1 ↑ |  |
| Within-group | Long-stay | Day 1 vs Day 14 | Bacteroidota | Bacteroidales | Porphyromonadaceae | Unclassified | 4.36 | 0.00 | 4.66 | Day 1 ↑ |  |
| Within-group | Long-stay | Day 1 vs Day 14 | Bacteroidota | Bacteroidales | Porphyromonadaceae | *Porphyromonas* | 4.36 | 0.00 | 4.66 | Day 1 ↑ |  |
| Within-group | Long-stay | Day 1 vs Day 14 | Firmicutes | Veillonellales-Selenomonadales | Veillonellaceae | *Negativicoccus* | 3.25 | 0.00 | 3.47 | Day 7 ↑ |  |
| Within-group | Long-stay | Day 1 vs Day 14 | Firmicutes | Lactobacillales | Streptococcaceae | Unclassified | 3.99 | 0.00 | 4.42 | Day 1 ↑ |  |
| Within-group | Long-stay | Day 1 vs Day 14 | Firmicutes | Peptostreptococcales-Tissierellales | norank_o__Peptostreptococcales-Tissierellales | *Fenollaria* | 4.31 | 0.00 | 4.53 | Day 1 ↑ |  |
| Within-group | Long-stay | Day 1 vs Day 14 | Firmicutes | Lactobacillales | Streptococcaceae | *Streptococcus* | 3.99 | 0.00 | 4.42 | Day 1 ↑ |  |
| Within-group | Long-stay | Day 1 vs Day 14 | Proteobacteria | Enterobacterales | Enterobacteriaceae | *Escherichia-Shigella* | 4.76 | 0.00 | 5.16 | Day 1 ↑ |  |
| Within-group | Long-stay | Day 1 vs Day 14 | Firmicutes | Christensenellales | Christensenellaceae | Unclassified | 3.76 | 0.00 | 4.20 | Day 1 ↑ |  |
| Within-group | Long-stay | Day 1 vs Day 14 | Firmicutes | Peptostreptococcales-Tissierellales | norank_o__Peptostreptococcales-Tissierellales | *Peptoniphilus* | 4.46 | 0.00 | 4.81 | Day 1 ↑ |  |
| Within-group | Long-stay | Day 1 vs Day 14 | Firmicutes | Unclassified | Unclassified | Unclassified | 5.16 | 0.00 | 5.50 | Day 1 ↑ |  |

Unclassified indicates taxa that could not be confidently assigned at the corresponding taxonomic level.

**Table S6**. **Differentially enriched taxa identified by LEfSe between ICU length-of-stay groups at matched time points. Taxa were selected using thresholds of LDA > 2.0 and p < 0.05.**

| **Comparison type** | **ICU group(s)** | **Time point(s)** | **Phylum** | **Order** | **Family** | **Genus** | **LDA score** | **p-value** | **Mean abundance** | **Enriched in** |
| --- | --- | --- | --- | --- | --- | --- | --- | --- | --- | --- |
| Between-group | Short vs Long | Day 1 | Proteobacteria | Burkholderiales | Sutterellaceae | *Sutterella* | 2.50 | 0.00 | 2.92 | Short ↑ |
| Between-group | Short vs Long | Day 1 | Actinobacteriota | Actinomycetales | Actinomycetaceae | *Actinotignum* | 2.58 | 0.00 | 1.91 | Short ↑ |
| Between-group | Short vs Long | Day 1 | Patescibacteria | Saccharimonadales | Saccharimonadaceae | Unclassified | 2.21 | 0.05 | 1.50 | Long ↑ |
| Between-group | Short vs Long | Day 1 | Spirochaetota | Spirochaetales | Spirochaetaceae | Unclassified | 2.55 | 0.03 | 2.13 | Short ↑ |
| Between-group | Short vs Long | Day 1 | Synergistota | Unclassified | Unclassified | Unclassified | 2.72 | 0.01 | 3.15 | Long ↑ |
| Between-group | Short vs Long | Day 1 | Firmicutes | Oscillospirales | Butyricicoccaceae | *UCG-009* | 2.67 | 0.02 | 1.78 | Short ↑ |
| Between-group | Short vs Long | Day 1 | Firmicutes | Oscillospirales | Oscillospiraceae | *UCG-005* | 3.34 | 0.03 | 3.69 | Short ↑ |
| Between-group | Short vs Long | Day 1 | Firmicutes | Oscillospirales | Oscillospiraceae | *UCG-003* | 2.44 | 0.02 | 2.78 | Short ↑ |
| Between-group | Short vs Long | Day 1 | Synergistota | Synergistales | Unclassified | Unclassified | 2.72 | 0.01 | 3.15 | Long ↑ |
| Between-group | Short vs Long | Day 1 | Firmicutes | Lachnospirales | Lachnospiraceae | *Lachnospiraceae_NK4A136_group* | 2.86 | 0.05 | 3.18 | Short ↑ |
| Between-group | Short vs Long | Day 1 | Actinobacteriota | Coriobacteriales | Eggerthellaceae | *norank_f__Eggerthellaceae* | 2.70 | 0.03 | 1.83 | Short ↑ |
| Between-group | Short vs Long | Day 1 | Spirochaetota | Unclassified | Unclassified | Unclassified | 2.75 | 0.03 | 2.13 | Short ↑ |
| Between-group | Short vs Long | Day 1 | Firmicutes | Christensenellales | Unclassified | Unclassified | 3.57 | 0.05 | 3.93 | Short ↑ |
| Between-group | Short vs Long | Day 1 | Firmicutes | Veillonellales-Selenomonadales | Veillonellaceae | *Megasphaera* | 3.27 | 0.01 | 3.66 | Short ↑ |
| Between-group | Short vs Long | Day 1 | Spirochaetota | Spirochaetales | Spirochaetaceae | *Treponema* | 2.47 | 0.03 | 2.13 | Short ↑ |
| Between-group | Short vs Long | Day 1 | Proteobacteria | Burkholderiales | Oxalobacteraceae | *Oxalobacter* | 3.06 | 0.05 | 1.07 | Short ↑ |
| Between-group | Short vs Long | Day 1 | Proteobacteria | Burkholderiales | Sutterellaceae | Unclassified | 2.84 | 0.00 | 3.29 | Short ↑ |
| Between-group | Short vs Long | Day 1 | Bacteroidota | Bacteroidales | Prevotellaceae | Unclassified | 4.44 | 0.04 | 5.12 | Short ↑ |
| Between-group | Short vs Long | Day 1 | Synergistota | Unclassified | Unclassified | Unclassified | 2.72 | 0.01 | 3.15 | Long ↑ |
| Between-group | Short vs Long | Day 1 | Bacteroidota | Bacteroidales | Prevotellaceae | *Prevotella* | 4.44 | 0.04 | 5.12 | Short ↑ |
| Between-group | Short vs Long | Day 1 | Firmicutes | Lactobacillales | Aerococcaceae | Unclassified | 3.26 | 0.04 | 3.71 | Short ↑ |
| Between-group | Short vs Long | Day 1 | Spirochaetota | Unclassified | Unclassified | Unclassified | 2.44 | 0.03 | 2.13 | Short ↑ |
| Between-group | Short vs Long | Day 1 | Firmicutes | Christensenellales | Christensenellaceae | *Christensenellaceae_R-7_group* | 3.57 | 0.05 | 3.93 | Short ↑ |
| Between-group | Short vs Long | Day 1 | Firmicutes | Oscillospirales | Oscillospiraceae | *NK4A214_group* | 2.26 | 0.05 | 2.78 | Short ↑ |
| Between-group | Short vs Long | Day 1 | Proteobacteria | Burkholderiales | Unclassified | Unclassified | 3.65 | 0.04 | 4.07 | Short ↑ |
| Between-group | Short vs Long | Day 1 | Synergistota | Synergistales | Synergistaceae | Unclassified | 2.72 | 0.01 | 3.15 | Long ↑ |
| Between-group | Short vs Long | Day 1 | Spirochaetota | Spirochaetales | Unclassified | Unclassified | 2.78 | 0.03 | 2.13 | Short ↑ |
| Between-group | Short vs Long | Day 1 | Firmicutes | Christensenellales | Christensenellaceae | Unclassified | 3.57 | 0.05 | 3.93 | Short ↑ |
| Between-group | Short vs Long | Day 1 | Proteobacteria | Burkholderiales | Oxalobacteraceae | Unclassified | 3.06 | 0.05 | 1.07 | Short ↑ |
| Between-group | Short vs Long | Day 7 | Actinobacteriota | Coriobacteriales | Eggerthellaceae | *Eggerthella* | 3.19 | 0.04 | 3.77 | Long ↑ |
| Between-group | Short vs Long | Day 7 | Firmicutes | Lachnospirales | Lachnospiraceae | *Lachnospiraceae_FCS020_group* | 2.54 | 0.02 | 2.01 | Short ↑ |
| Between-group | Short vs Long | Day 7 | Firmicutes | Lachnospirales | Lachnospiraceae | *Ruminococcus_gauvreauii_group* | 2.25 | 0.00 | 2.46 | Short ↑ |
| Between-group | Short vs Long | Day 7 | Actinobacteriota | Unclassified | Unclassified | Unclassified | 4.24 | 0.04 | 4.80 | Long ↑ |
| Between-group | Short vs Long | Day 7 | Actinobacteriota | Corynebacteriales | Corynebacteriaceae | Unclassified | 3.81 | 0.04 | 4.23 | Long ↑ |
| Between-group | Short vs Long | Day 7 | Proteobacteria | Enterobacterales | Enterobacteriaceae | *Citrobacter* | 2.92 | 0.05 | 3.27 | Long ↑ |
| Between-group | Short vs Long | Day 7 | Actinobacteriota | Corynebacteriales | Corynebacteriaceae | *Corynebacterium* | 3.74 | 0.03 | 4.11 | Long ↑ |
| Between-group | Short vs Long | Day 7 | Proteobacteria | Rhizobiales | Unclassified | Unclassified | 2.59 | 0.05 | 1.37 | Long ↑ |
| Between-group | Short vs Long | Day 7 | Patescibacteria | Saccharimonadales | Saccharimonadaceae | *TM7x* | 3.41 | 0.03 | 1.06 | Short ↑ |
| Between-group | Short vs Long | Day 7 | Firmicutes | Lactobacillales | Lactobacillaceae | *Lactobacillus* | 4.55 | 0.01 | 4.90 | Short ↑ |
| Between-group | Short vs Long | Day 7 | Firmicutes | Staphylococcales | Gemellaceae | *Gemella* | 4.16 | 0.05 | 1.36 | Short ↑ |
| Between-group | Short vs Long | Day 7 | Firmicutes | Lactobacillales | Lactobacillaceae | Unclassified | 4.55 | 0.01 | 4.90 | Short ↑ |
| Between-group | Short vs Long | Day 7 | Firmicutes | Oscillospirales | Ruminococcaceae | *Fournierella* | 3.04 | 0.05 | 3.29 | Long ↑ |
| Between-group | Short vs Long | Day 7 | Firmicutes | Staphylococcales | Gemellaceae | Unclassified | 4.18 | 0.05 | 1.36 | Short ↑ |

Unclassified indicates taxa that could not be confidently assigned at the corresponding taxonomic level.

**Table S7.** **Clinical Characteristics of OTU502- and OTU967-Dominated Subgroups Among Long-Stay ICU Patients on Day 7**

| **Variable** | **OTU502 (n = 8)** | **OTU967 (n = 15)** | **p-value** |
| --- | --- | --- | --- |
| **Demographics** |  |  |  |
| Sex (male), n (%) | 5 (62.5) | 10 (66.7) | >0.999 |
| Age, years | 32.5 ± 15.2 | 48.4 ± 15.6 | 0.030^*^ |
| BMI, kg/m² | 22.6 ± 3.7 | 24.0 ± 2.2 | 0.272 |
| Hypertension, n (%) | 2 (25.0) | 6 (40.0) | 0.657 |
| Diabetes mellitus, n (%) | 1 (12.5) | 2 (13.3) | >0.999 |
| **Clinical Diagnosis and Surgery** |  |  |  |
| Admitting diagnosis (Supratentorial / Infratentorial / Other), n (%) | 2 (25.0) / 6 (75.0) / 0 (0.0) | 4 (26.7) / 9 (60.0) / 2 (13.3) | 0.483 |
| Operative duration, h | 5.9 ± 1.6 | 8.7 ± 3.7 | 0.065 |
| Estimated blood loss, mL | 300 (300–1775) | 1000 (400–1200) | 0.205 |
| Intraoperative hypotension duration, min | 15.0 (0–33.75) | 0 (0–20) | 0.085 |
| Postoperative complications, n (%) | 3 (37.5) | 8 (53.3) | 0.669 |
| Postoperative hypoalbuminemia (Normal / Mild / Moderate / Severe), n (%) | 5 (62.5) / 2 (25.0) / 0 (0.0) / 1 (12.5) | 8 (53.3) / 6 (40.0) / 1 (6.7) / 0 (0.0) | 0.877 |
| **Severity and Neurological Scores** |  |  |  |
| APACHE II score at ICU admission | 14.0 ± 4.1 | 16.3 ± 4.5 | 0.246 |
| Lowest GCS during ICU stay | 9 (8–10) | 10 (5–10) | 0.511 |
| GCS at discharge | 14 (9–14) | 13 (6–13) | 0.062 |
| **Therapies and ICU Exposure** |  |  |  |
| Vasopressor therapy duration, h | 0 (0–34.5) | 0 (0–0) | 0.924 |
| Antibiotic therapy duration, days | 14 (14–14) | 14 (13–14) | 0.353 |
| Probiotic use duration, days | 3.5 (0–12.8) | 0 (0–6) | 0.162 |
| PPI use duration, days | 14 (11.8–14) | 14 (13–14) | 0.924 |
| Prokinetic use duration, days | 0 (0–4.5) | 0 (0–0) | 0.079 |
| Mucosal protectant use duration, days | 0 (0–3) | 0 (0–7) | 0.427 |
| Enteral nutrition formula (Peptide-based / Standard / High-calorie / Diabetes-specific / None), n (%) | 3 (37.5) / 1 (12.5) / 3 (37.5) / 0 (0.0) / 1 (12.5) | 5 (33.3) / 3 (20.0) / 5 (33.3) / 2 (13.3) / 0 (0.0) | 0.350 |
| **Gastrointestinal Symptoms** |  |  |  |
| Constipation, n (%) | 6 (75.0) | 15 (100.0) | 0.111 |
| Diarrhea, n (%) | 2 (25.0) | 3 (20.0) | 0.182 |
| Upper GI bleeding, n (%) | 2 (25.0) | 6 (40.0) | 0.657 |
| Abdominal bloating, n (%) | 4 (50.0) | 6 (40.0) | 0.685 |
| Gastric retention, n (%) | 3 (37.5) | 8 (53.3) | 0.667 |
| Vomiting, n (%) | 1 (12.5) | 3 (20.0) | 0.694 |
| **ICU Outcomes** |  |  |  |
| Mechanical ventilation duration, days | 8.4 ± 5.2 | 5.7 ± 5.3 | 0.242 |
| Final airway management (Extubated / Artificial airway in place), n (%) | 6 (75.0) / 2 (25.0) | 13 (86.7) / 2 (13.3) | 0.589 |
| ICU length of stay, days | 18.5 (15.5–29.8) | 20 (14–26) | 0.762 |
| ICU mortality, n (%) | 0 (0.0) | 1 (6.7) | >0.999 |
| Values are presented as mean ± standard deviation or median (interquartile range), as appropriate. Between-group comparisons were performed using the Mann–Whitney U test for continuous variables and the Fisher’s exact test for categorical variables. Subgroup classification was determined independently at each sampling time point based on the dominant OTU; therefore, subgroup membership could change between Day 7 and Day 14. * p < 0.05. | | | |
|  |  |  |  |

**Table S8. Clinical Characteristics of OTU502- and OTU967-Dominated Subgroups Among Long-Stay ICU Patients on Day 14**

| **Variable** | **OTU502 (n = 15)** | **OTU967 (n = 8)** | **p-value** |
| --- | --- | --- | --- |
| **Demographics** |  |  |  |
| Sex (male), n (%) | 10 (66.7) | 5 (62.5) | >0.999 |
| Age, years | 43.1 ± 18.9 | 42.5 ± 13.9 | 0.941 |
| BMI, kg/m² | 23.3 ± 3.0 | 24.0 ± 2.6 | 0.572 |
| Hypertension, n (%) | 5 (33.3) | 3 (37.5) | >0.999 |
| Diabetes mellitus, n (%) | 2 (13.3) | 1 (12.5) | 0.957 |
| **Clinical Diagnosis and Surgery** |  |  |  |
| Admitting diagnosis (Supratentorial / Infratentorial / Other), n (%) | 4 (26.7) / 9 (60.0) / 2 (13.3) | 2 (25.0) / 6 (75.0) / 0 (0.0) | 0.429 |
| Operative duration, h | 7.2 ± 3.4 | 8.6 ± 3.4 | 0.296 |
| Estimated blood loss, mL | 500 (300–1000) | 750 (300–2700) | 0.788 |
| Intraoperative hypotension duration, min | 0 (0–30) | 0 (0–7.5) | 0.187 |
| Postoperative complications, n (%) | 0 (0.0) | 1 (12.5) | >0.999 |
| Postoperative hypoalbuminemia (Normal / Mild / Moderate / Severe), n (%) | 8 (53.3) / 5 (33.3) / 1 (6.7) / 1 (6.7) | 5 (62.5) / 3 (37.5) / 0 (0.0) / 0 (0.0) | 0.567 |
| **Severity and Neurological Scores** |  |  |  |
| APACHE II score at ICU admission | 15.7 ± 4.5 | 15.0 ± 4.3 | 0.711 |
| Lowest GCS during ICU stay | 10 (6–10) | 8 (7–10) | 0.450 |
| GCS at discharge | 13 (6–14) | 13 (8–13) | 0.573 |
| **Therapies and ICU Exposure** |  |  |  |
| Vasopressor therapy duration, h | 0 (0–0) | 0 (0–47.2) | 0.227 |
| Antibiotic therapy duration, days | 14 (13–14) | 14 (13.1–14) | >0.999 |
| Probiotic use duration, days | 3 (0–9) | 0 (0–0) | 0.084 |
| PPI use duration, days | 14 (13–14) | 14 (13.3–14) | >0.999 |
| Prokinetic use duration, days | 0 (0–0) | 0 (0–0) | >0.999 |
| Mucosal protectant use duration, days | 0 (0–3) | 1 (0–6.3) | 0.329 |
| Enteral nutrition formula (Peptide-based / Standard / High-calorie / Diabetes-specific / None), n (%) | 4 (26.7) / 3 (20.0) / 6 (40.0) / 2 (13.3) / 0 (0.0) | 4 (50.0) / 1 (12.5) / 2 (25.0) / 1 (12.5) / 0 (0.0) | 0.081 |
| **Gastrointestinal Symptoms** |  |  |  |
| Constipation, n (%) | 14 (93.3) | 7 (87.5) | >0.999 |
| Diarrhea, n (%) | 6 (40.0) | 1 (12.5) | 0.345 |
| Upper GI bleeding, n (%) | 4 (26.7) | 4 (50.0) | 0.371 |
| Abdominal bloating, n (%) | 7 (46.7) | 3 (37.5) | >0.999 |
| Gastric retention, n (%) | 6 (40.0) | 5 (62.5) | 0.400 |
| Vomiting, n (%) | 2 (13.3) | 2 (25.0) | 0.589 |
| **ICU Outcomes** |  |  |  |
| Mechanical ventilation duration, days | 5 (0–11) | 8.5 (4–13.4) | 0.143 |
| Final airway management (Extubated / Artificial airway in place), n (%) | 11 (73.3) / 4 (26.7) | 8 (100.0) / 0 (0.0) | 0.237 |
| ICU length of stay, days | 20 (15–30) | 23 (17.8–26) | 0.577 |
| ICU mortality, n (%) | 1 (6.7) | 0 (0.0) | >0.9999 |
| Values are presented as mean ± standard deviation or median (interquartile range), as appropriate. Between-group comparisons were performed using the Mann–Whitney U test for continuous variables and the Fisher’s exact test for categorical variables. Subgroup classification was determined independently at each sampling time point based on the dominant OTU; therefore, subgroup membership could change between Day 7 and Day 14. | | | |

**Table S9. Predicted microbial phenotypes inferred by BugBase.**

| **Phenotype** | **Group 1** | **Group 2** | **Group 1 mean** **(%)** | **Group 1 SD (%)** | **Group 2 mean (%)** | **Group 2 SD (%)** | **p-value** | **FDR-adjusted p-value** |
| --- | --- | --- | --- | --- | --- | --- | --- | --- |
| **Stress tolerant** | Long-Stay | Short-Stay | 26.74 | 2.49 | 27.92 | 1.9 | 0.15 | 0.54 |
| **Anaerobic** | Long-Stay | Short-Stay | 22.37 | 5.23 | 20.15 | 5.72 | 0.34 | 0.54 |
| **Gram-positive** | Long-Stay | Short-Stay | 19.08 | 6.48 | 20.67 | 6.55 | 0.48 | 0.54 |
| **Potentially pathogenic** | Long-Stay | Short-Stay | 9.68 | 4.76 | 8.41 | 4.67 | 0.56 | 0.56 |
| **Gram-negative** | Long-Stay | Short-Stay | 9.1 | 5.7 | 7.6 | 5.15 | 0.47 | 0.54 |
| **Contains mobile elements** | Long-Stay | Short-Stay | 4.97 | 5.83 | 7.77 | 7.75 | 0.38 | 0.54 |
| **Forms biofilms** | Long-Stay | Short-Stay | 4.78 | 3.24 | 4.5 | 4.3 | 0.48 | 0.54 |
| **Aerobic** | Long-Stay | Short-Stay | 2.02 | 2.06 | 2.01 | 4.19 | 0.22 | 0.54 |
| **Facultatively anaerobic** | Long-Stay | Short-Stay | 1.25 | 1.08 | 0.98 | 0.88 | 0.31 | 0.54 |
| **Stress tolerant** | OTU502-High | OTU502-Low | 27.82 | 2.04 | 26.61 | 2.49 | 0.17 | 0.72 |
| **Anaerobic** | OTU502-High | OTU502-Low | 21.53 | 5.67 | 21.4 | 5.43 | 0.99 | 0.99 |
| **Gram-positive** | OTU502-High | OTU502-Low | 20.87 | 6.59 | 18.56 | 6.35 | 0.32 | 0.72 |
| **Potentially pathogenic** | OTU502-High | OTU502-Low | 8.45 | 4.79 | 9.87 | 4.69 | 0.64 | 0.93 |
| **Gram-negative** | OTU502-High | OTU502-Low | 7.36 | 5.13 | 9.65 | 5.73 | 0.27 | 0.72 |
| **Contains mobile elements** | OTU502-High | OTU502-Low | 6.35 | 7.1 | 5.93 | 6.55 | 0.83 | 0.94 |
| **Forms biofilms** | OTU502-High | OTU502-Low | 4.58 | 4.11 | 4.74 | 3.27 | 0.55 | 0.93 |
| **Aerobic** | OTU502-High | OTU502-Low | 1.99 | 3.79 | 2.05 | 2.15 | 0.29 | 0.72 |
| **Facultatively anaerobic** | OTU502-High | OTU502-Low | 1.05 | 0.9 | 1.2 | 1.08 | 0.73 | 0.93 |

Values are presented as mean ± standard deviation (%). No significant differences were observed after FDR correction.
